# Cefuroxime axetil dosing regimens and probability of target attainment in adults and children

**DOI:** 10.1002/bcp.70158

**Published:** 2025-07-17

**Authors:** Sven C. van Dijkman, Praveen Kamble, Jerzy A. Kowalski, Oscar Della Pasqua

**Affiliations:** ^1^ GSK Clinical Pharmacology Modelling and Simulation London UK; ^2^ GSK Global Medical Affairs Worli Mumbai Maharashtra India; ^3^ GSK Clinical Safety and Pharmacovigilance Warsaw Poland; ^4^ Clinical Pharmacology & Therapeutics Group University College London London UK

**Keywords:** bacterial infections, bacterial susceptibility, cefuroxime, dose rationale, probability of target attainment

## Abstract

**Aims:**

Cefuroxime axetil exists in several dosage forms for oral administration, and is indicated for treatment of respiratory, genitourinary, skin and soft tissue infections. Evolving patterns of bacterial susceptibility, expressed as increasing minimum inhibitory concentrations (MICs), warrant monitoring of antibiotic efficacy. Here we investigate the performance of different cefuroxime axetil doses required to yield the desired target exposure against the predominant pathogens for each indication.

**Methods:**

The pharmacokinetics of cefuroxime in plasma/serum and urine was characterized after oral, intravenous and intramuscular administration. Covariates included the effect of weight on clearance and volume of distribution, and formulation on absorption parameters. Subsequently, systemic cefuroxime concentrations over time were simulated to assess the time above the MIC (T > MIC) and the probability of target attainment (PTA) for different dosing regimens in adults and children.

**Results:**

Tablet doses of 250 and 500 mg twice daily achieved the target T > MIC (40%) and PTA (≥90%) for MICs up to 0.25 and 0.5 mg/L, respectively, thereby covering most European Committee on Antimicrobial Susceptibility Testing breakpoints for key pathogens. Due to its absorption profile, the oral suspension covers even higher MICs, up to 1.0 mg/L. As cefuroxime is mostly excreted unchanged in urine, cefuroxime axetil doses of 250 mg twice daily for urinary tract infection yield a PTA of 100% for MIC values up to 8 mg/L.

**Conclusions:**

Our analysis provides insight into the performance of different doses and dosing regimens for cefuroxime axetil for key pathogens. As resistance patterns evolve, and differ between countries or regions, cefuroxime doses may require adjustments considering local bacterial susceptibility.

## Graphical abstract

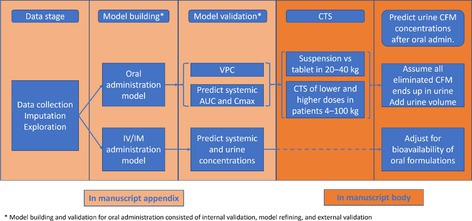

**What is already known about this subject**

- Cefuroxime is a second‐generation cephalosporin with enhanced activity against Gram‐negative bacteria.
- Cefuroxime has been extensively utilized for the treatment of respiratory, digestive, urinary, musculoskeletal, skin and soft tissue infections. Currently, it is on the World Health Organization’s list of essential medicines.
- Cefuroxime has excellent activity against Gram‐positive streptococci and Gram‐negative aerobes, but information is lacking on the performance of different doses and dosing regimens when prescribing it to patients.
- Understanding of differences in the probability of target attainment is essential to prevent treatment failure and counteract the development of bacterial resistance.

**What this study adds**

- Historical data were leveraged to develop a pharmacokinetic model that adequately reflects cefuroxime exposure as well as interindividual variation in adult and paediatric subjects.
- The availability of a pharmacokinetic model enabled the determination of time above minimum inhibitory concentration and probability of target attainment for various dosing regimen and weight categories.
- Given the large variation in bacterial susceptibility across regions and time, the current investigation provides a starting point for dosing decisions made by prescribers.

## 1 INTRODUCTION

Cefuroxime axetil, an orally administered prodrug of cefuroxime, is absorbed from the gastrointestinal tract and hydrolysed by nonspecific esterases into the active form cefuroxime.^1^ Cefuroxime exerts its bactericidal effect by attachment to penicillin‐binding proteins, inhibiting bacterial cell wall synthesis.^1^ Cefuroxime axetil is indicated for upper/lower respiratory tract, genitourinary tract, skin and soft tissue infections, gonorrhoea, treatment of early‐ and subsequent prevention of late‐Lyme disease.^2^

Besides a tablet formulation, cefuroxime axetil is available as suspension for use in paediatric patients and those unable/unwilling to swallow tablets. Previous investigations showed that this suspension results in ≤17% lower systemic exposure (the area under the concentration*–*time curve [AUC]) compared with tablets, presumably due to reduced bioavailability.^3^ However, at similar dose levels the suspension's slower absorption rate results in longer overall absorption time, with slightly lower peak concentrations (Cmax), but similar time above the minimum inhibitory concentrations (T > MIC) *vs*. tablets.^3^ Typical cefuroxime axetil doses in adults and children weighing >40 kg are 250 or 500 mg twice daily (BID), depending on the infection. For patients weighing ≤40 kg, typical suspension doses are 10 or 15 mg/kg BID depending on the infection, capped to a maximum of 500 or 1000 mg total daily dose, respectively.^4^

**FIGURE 1:**
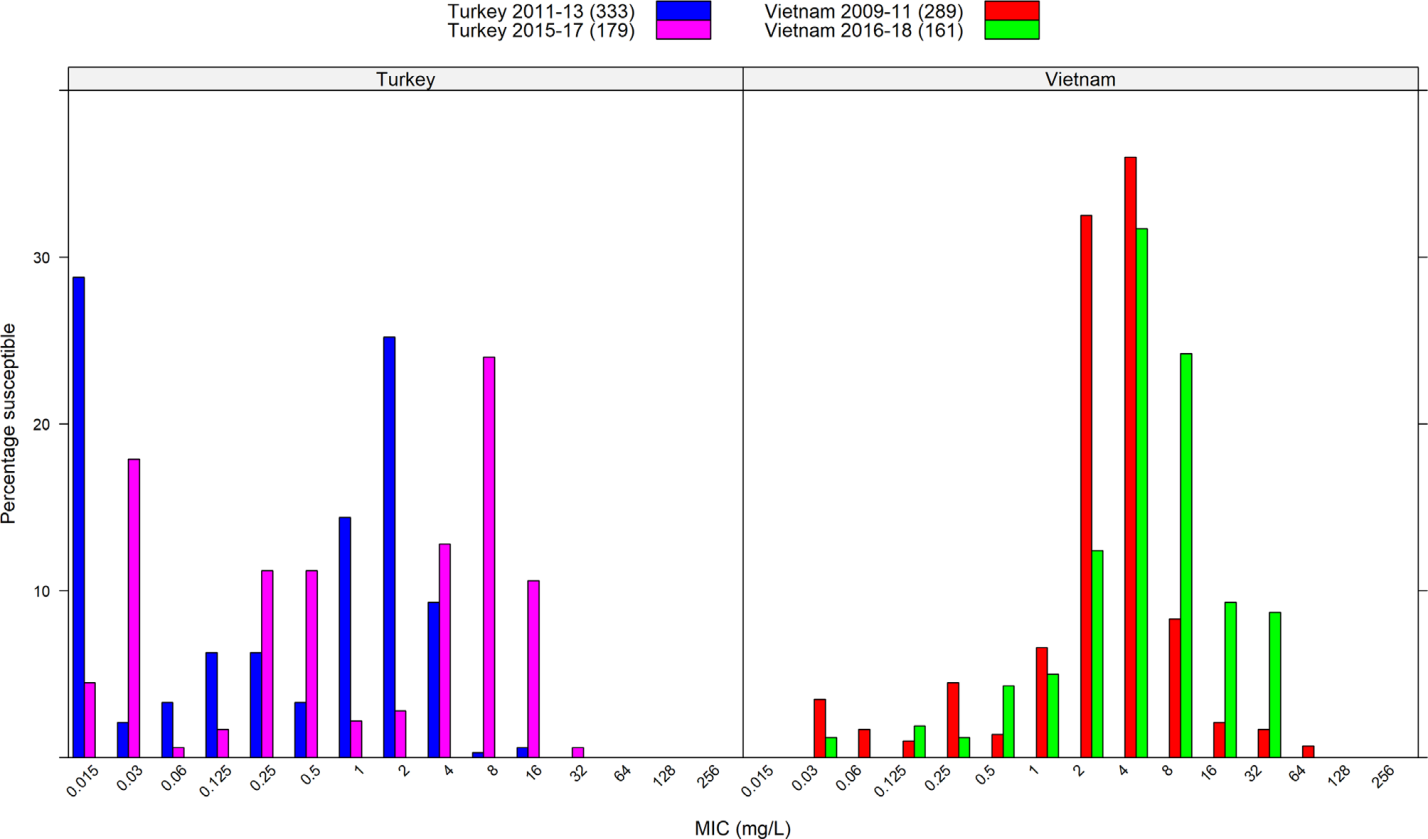
Histograms show geographical and temporal differences in cefuroxime MIC distribution for *Streptococcus pneumoniae* isolates obtained from SOAR data in Turkey and Vietnam over a 2‐year period.^5^, ^6^, ^7^, ^8^ MIC, minimum inhibitory concentration; SOAR, survey of antimicrobial resistance.

The susceptibility of microbial pathogens to antibiotic agents differs across regions, and may evolve over time due to variable prescription practices and adherence. The survey of antimicrobial resistance (SOAR) tracked the susceptibility of pathogens such as *Streptococcus pneumoniae* and *Haemophilus influenzae* to several antibiotics, including cefuroxime. Examples of published SOAR data are presented in Figure 1,^5^, ^6^, ^7^, ^8^ showing the distribution of sampled MICs for cefuroxime, over two time periods in Turkey and Vietnam. Over time, the MIC distribution right‐shifted (worsened) for both countries, albeit seemingly at different rates. Even though Figure 1 represents a small sample and may be subject to confounding, its variation does reflect temporal and geographical differences,^9^, ^10^, ^11^ aligning with a trend observed in other SOAR data. Given this evolving pattern in the susceptibility of microbial pathogens, we assessed the ability of cefuroxime axetil doses to achieve adequate T> MIC and PTA across indications for the key pathogens (e.g. *S. pneumoniae, E* *scherichia coli* *)*, taking into account the differences in formulations. While cefuroxime axetil prescription should consider local guidance and susceptibility patterns, the current study aims to provide the prescriber with data to support such decision making. One established method for the evaluation of the PTA is to employ a pharmacokinetic (PK) model and determine T > MIC and PTA based on simulated concentration‐over‐time profiles for various dosing scenarios, as previously implemented for different antibiotics and pathogens (e.g. piperacillin/tazobactam, rifampicin^12^, ^13^).

## 2 METHODS

An overview of the steps involved in data selection, model building, model validation, clinical trial simulations and prediction of cefuroxime concentrations along with the corresponding PTA for the different dosage forms and regimens is provided in Figure S1 (Supporting Information).

### 2.1 Data, model building and validation

Model development and validation were based on summary data extracted from 4 publications: Donn *et al*., Foord, Ginsburg *et al*., and Powell *et al*.^3^, ^14^, ^15^, ^16^ These publications provided the necessary data for further PK characterization of both dosage forms, at different dose levels across a wide group of individuals (i.e. healthy volunteers and patients), including paediatric and adult patients, with systemic and urine exposure information. Ginsburg *et al*.^15^ measured drug concentrations in plasma, the other publications^3^, ^14^, ^16^ measured serum concentrations. Foord^14^ also measured urine cefuroxime. Overall, the data collected represented 139 individuals with 179 plasma and serum samples and 49 urine samples, covering ages 3 months–57 years and weights 5–94 kg (Table S1).

Population PK model building followed standard PK compartmental analysis methods, including drug excretion in urine, as previously described.^17^, ^18^ Additionally, a time‐dependent absorption constant was modelled in line with previous findings.^19^ Due to limitations of using summary data, weight and age were based on averages reported in the respective literature sources, or imputed where necessary (Section S2 and Table S2). This limitation also required a priori allometric scaling to ensure appropriate weight adjustment.^20^ After inclusion of allometry, any remaining correlations between covariates and interindividual variation (IIV) were evaluated graphically, and tested against relevant parameters if a trend was observed. Model validation used visual predictive checks, comparing simulated/observed Cmax and AUC values. Difficulties in combining data from administration of intravenous (IV) and intramuscular (IM) doses with tablet and suspension doses required a separate model for these subsets (Sections S3.2 and S3.3).

### 2.2 Clinical trial simulations

Simulation scenarios were implemented using the final model describing cefuroxime concentrations after oral administration of tablets and suspension, as well as the final model describing the amount excreted in urine after IV and IM administration. In these, the effect of dose, dosing regimen, formulation and covariate factors (i.e. IIV due to body weight) can be characterized in an integrated manner.^12^, ^13^, ^21^ The use of simulation scenarios as a tool for dose optimisation is well‐established, widely accepted both in clinical and regulatory contexts.^22^, ^23^ Here, simulation scenarios were evaluated to answer the following questions:

(i) Does switching from suspension to tablet form or vice versa have a clinically relevant effect on the target exposure (and T>MIC for the therapeutic targets) in patients weighing 20–40 kg (scenario 1)?

(ii) Do currently recommended paediatric and adult doses sufficiently cover the susceptibility (MIC) levels for key pathogens associated with the current indications for cefuroxime axetil?

This second question was answered in 2 separate simulation scenarios: one for urine exposure in the case of urinary tract infections (UTIs; MIC ≤8 mg/L, scenario 3) and another for systemic exposure in all other indications (MIC ≤0.5 mg/L, scenario 2).

#### 2.2.1 Virtual patient cohorts

**FIGURE 2:**
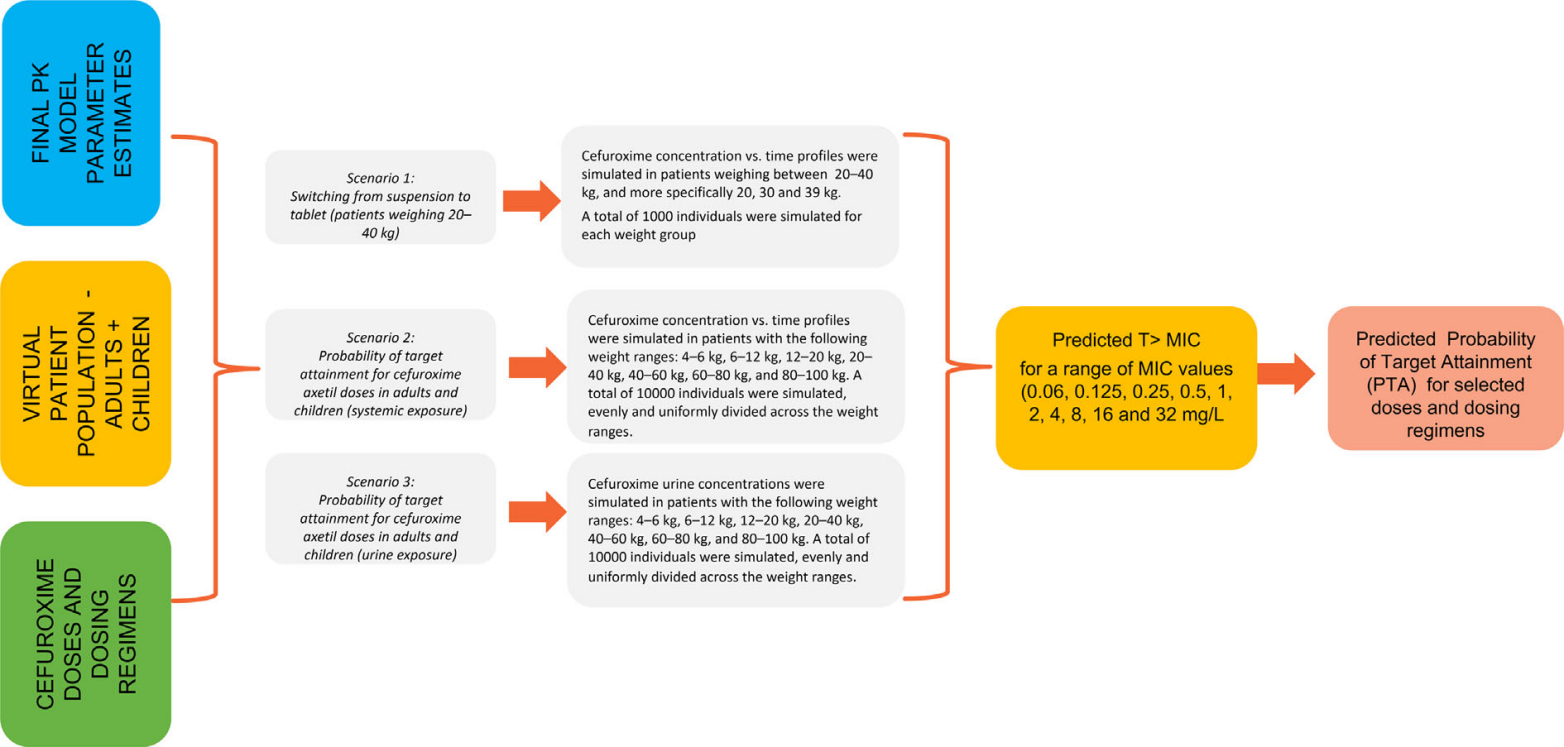
Simulation scenarios describing the concentration–time profiles of cefuroxime and the corresponding PTA in plasma/serum (scenarios 1 and 2) or urine (scenario 3) following switching from suspension to tablet or low and high doses of cefuroxime axetil. Even though the usual course of therapy may range from five to ten days, simulations were based on a single dose assuming no accumulation at steady state. PK, pharmacokinetics; PTA, probability of target attainment; T > MIC, time above minimum inhibitory concentration.

Cefuroxime plasma and urine concentration*–*time profiles following BID oral administration were simulated at 0.5‐h intervals over the course of 24 h. As cefuroxime does not accumulate, single‐dose PK data in plasma and urine was assumed to describe steady‐state conditions equally well. For scenario 1, 1000 virtual patients were simulated per weight category. For scenarios 2 and 3, 10000 virtual patients were simulated per dose level and formulation, equally distributed across weight ranges describing the adult and paediatric population ≥ 3 months old. Weight was simulated uniformly within each weight group (20, 30, 39 kg for scenario 1; 4–<6, 6–<12, 12–<20, 20–<40, 40–<60, 60–<80, 80–<100 kg for scenarios 2 and 3). The weight ranges for scenarios 2 and 3 were chosen to approximate different paediatric age groups of 4–<6 kg (0.25–1 years), 6–<12 kg (1–2 years), 12–<20 kg (2–4 years), 20–<40 kg (4–11 years), 40–<60 kg (>12 years), 60–<80 kg (>16 years) and 80–<100 kg (>16 years). As weight was the only covariate included in the PK model, no further baseline demographic characteristics were simulated. All individuals were assumed to have normal renal function. Doses were based on the current dosing guidance, i.e., 250 mg BID (lower dose) or 500 mg BID (higher dose) as tablets for patients >40 kg and 10 mg/kg BID (lower dose) or 15 mg/kg BID (higher dose) as suspension for patients ≤40 kg. In the case of switching from suspension (i.e., dose calculated in mg/kg) to tablet, doses were rounded to the nearest whole tablet (Table S6). The different simulation scenarios are depicted in Figure 2.

#### 2.2.2 Evaluation of T > MIC and PTA

Simulated cefuroxime concentration*–*time profiles from each scenario, obtained after each dosing regimen, were compared against a range of MICs (0.06, 0.125, 0.25, 0.5, 1, 2, 4, 8, 16, 32 mg/L). This allowed assessment of the T > MIC for each individual in the virtual cohort. To calculate T > MIC, the percentage of simulated concentrations across the simulated interval >MIC was determined for each individual virtual patient. Subsequently, the proportion (percentage) of individuals within each cohort achieving T > MIC ≥40% for each MIC, i.e., the PTA was calculated for each dosing regimen. In addition, the parameters of interest (AUC, Cmax, T > MIC, PTA) calculated for each treatment condition/scenario were summarized using descriptive statistics and graphically, as PTA *vs*. MIC graphs, stratified for dose and weight category.

#### 2.2.3 Statistical analysis

Simulations, summary statistics and graphical representations were all performed in statistical software R v3.2.5 and C++, with packages data, Table, Rcpp and lattice. Standard errors of model parameters during model building could only be obtained by covariance matrix and not by bootstrapping due to the relatively low number of individual PK profiles. No formal hypothesis testing was performed when comparing T > MIC or PTA between doses or formulations. Instead, we focussed on ensuring that the simulated doses were able to cover the appropriate MIC for the relevant pathogens under the assumption that the large number of virtual patients sufficiently accounted for IIV.

## 3 RESULTS

### 3.1 Model building and validation

In brief, two PK models have been developed, which adequately described the mean cefuroxime concentration–time profiles in plasma/serum and urine for each of the dosage forms and dosing regimens (tablet, suspension, IV and IM doses at different dose levels), taking into account the differences in bioavailability of the dosage forms. All parameters were estimated with acceptable precision and validation steps confirmed the appropriateness of the models for simulation purposes (Table S3 and Table S5). A singular model could not be built, possibly due to numerical difficulties in reconciliating the sharp rise in concentrations after IM or IV administration *vs*. the slower rise after oral administration. Variability in systemic exposure was characterized by estimates of IIV in the absorption rate constant, volume of distribution and clearance. Even though these estimates reflected intergroup and interstudy differences, final model estimates adequately replicated the variability in AUC and Cmax within each subgroup across the different studies (Table S4). With the a priori inclusion of allometry, no further covariates could be identified, including the potential effect of maturation processes describing renal function in infants and very young children. Given the adequate prediction of AUC and Cmax from the original studies across age groups, the inclusion of allometry was deemed sufficient to describe the changes in cefuroxime disposition in young children.

### 3.2 PTA

#### 3.2.1 Scenario 1: switching from suspension to tablet in patients 20–40 kg

**FIGURE 3:**
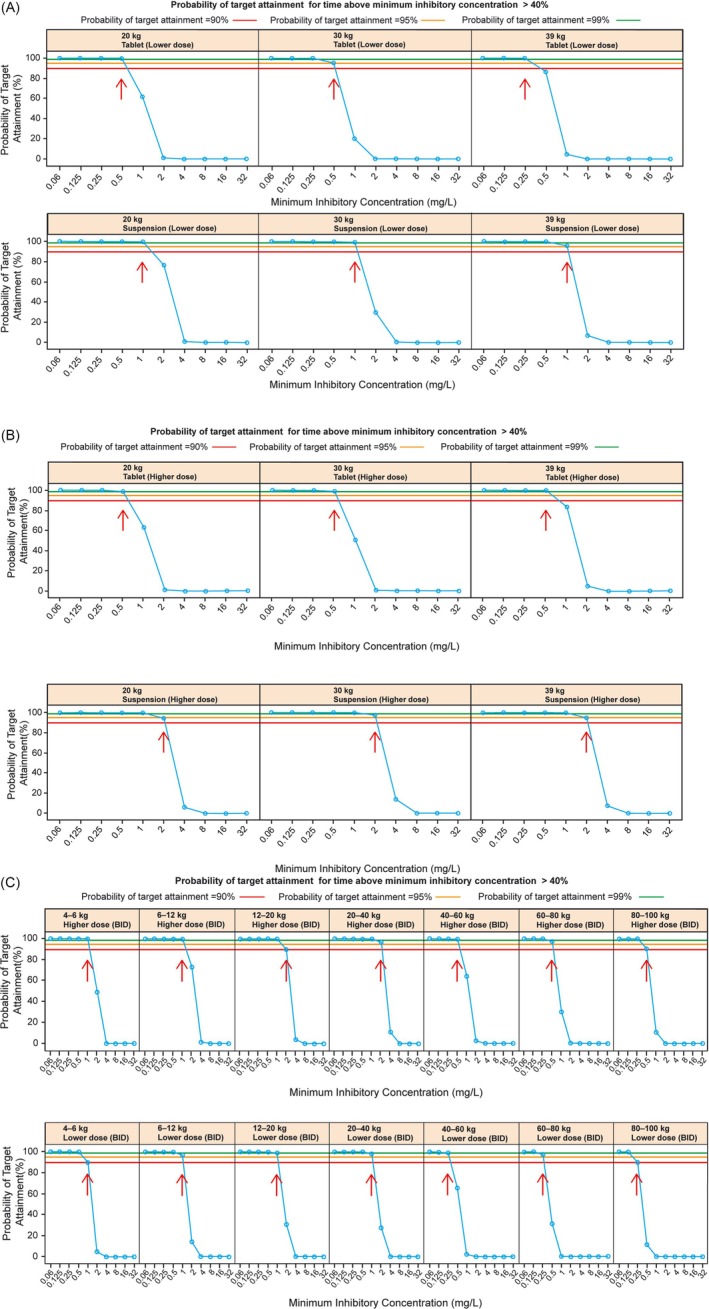
Panels A and B show the PTA in serum following administration of cefuroxime axetil doses of 10‐mg/kg and 15‐mg/kg, as tablet or suspension to paediatric patients stratified by body weight. Panel C shows the PTA in serum following administration of lower or higher doses of cefuroxime axetil as tablet or suspension to paediatric and adult patients stratified by body weight. Blue circles and lines denote the PTA at each MIC. Red, orange and green lines provide reference for 90%, 95% and 99% PTA, respectively. A *lower dose* corresponds to 10 mg/kg BID in patients ≤40 kg and 250 mg BID in those >40 kg. A *higher dose* corresponds to 15 mg/kg BID in patients ≤40 kg and 500 mg BID in patients >40 kg. Red arrows are shown at the highest MIC at which the PTA is ≥ 90%. BID, twice daily; MIC, minimum inhibitory concentration; PTA, probability of target attainment.

Simulated exposure (AUC, Cmax), T > MIC and PTA across the weight and dose cohorts are summarized in Tables S8, S9 and S10, respectively. The PTA associated with profiles after administration of the lower dose (10 mg/kg) in paediatric patients weighing 20, 30 and 39 kg show that both tablet and suspension formulations provide antibacterial cover (PTA of at least 90%) for MICs ≤0.25 mg/L, with tablets achieving cover for MICs ≤0.5 mg/L in those of 20 and 30 kg, and suspension achieving cover for infections with MICs ≤1.0 mg/L (Figure 3A). Conversely, the PTA associated with the higher dose (15 mg/kg) provides cover for higher MICs, which in the case of suspension reaches 2.0 mg/L, and ≤0.5 mg/L in the case of tablets (Figure 3B). Despite evidence of differences in the PTA between suspension and tablets, both formulations offer appropriate antibacterial cover for pathogens with MICs ≤0.5 mg/L.

#### 3.2.2 Scenario 2: PTA for cefuroxime axetil doses in adults and children (systemic exposure)

Simulated exposure (AUC, Cmax), T > MIC and PTA across different weight bands and dose cohorts are summarized in Tables S11, S12 and S13, respectively. The lower doses of cefuroxime axetil (10 mg/kg BID in patients ≤40 kg and 250 mg BID in patients >40 kg) appear to be appropriate (PTA of at least 90%) for the treatment of infections caused by pathogens with an MIC ≤0.25 mg/L (Figure 3C, lower row). This lower dose is typically used in easy‐to‐treat infections, where MIC tends to be well below that threshold, such as *Streptococcus pyogenes* (group A β‐haemolytic streptococci) with mean MIC_90_ values ≤0.25 mg/L.^1^ By contrast, higher doses of cefuroxime (15 mg/kg BID in patients ≤40 kg and 500 mg BID in patients >40 kg) are appropriate for the treatment of infections caused by pathogens with an MIC ≤0.5 mg/L (Figure 3C, upper row). Results show, however, that adequate exposures are likely to be achieved in patients weighing up to 100 kg. As the data available for model building did not include obese individuals, these results cannot be extrapolated to a wider weight range.

#### 3.2.3 Scenario 3: PTA for cefuroxime axetil doses in adults and children (urine exposure)

**FIGURE 4:**
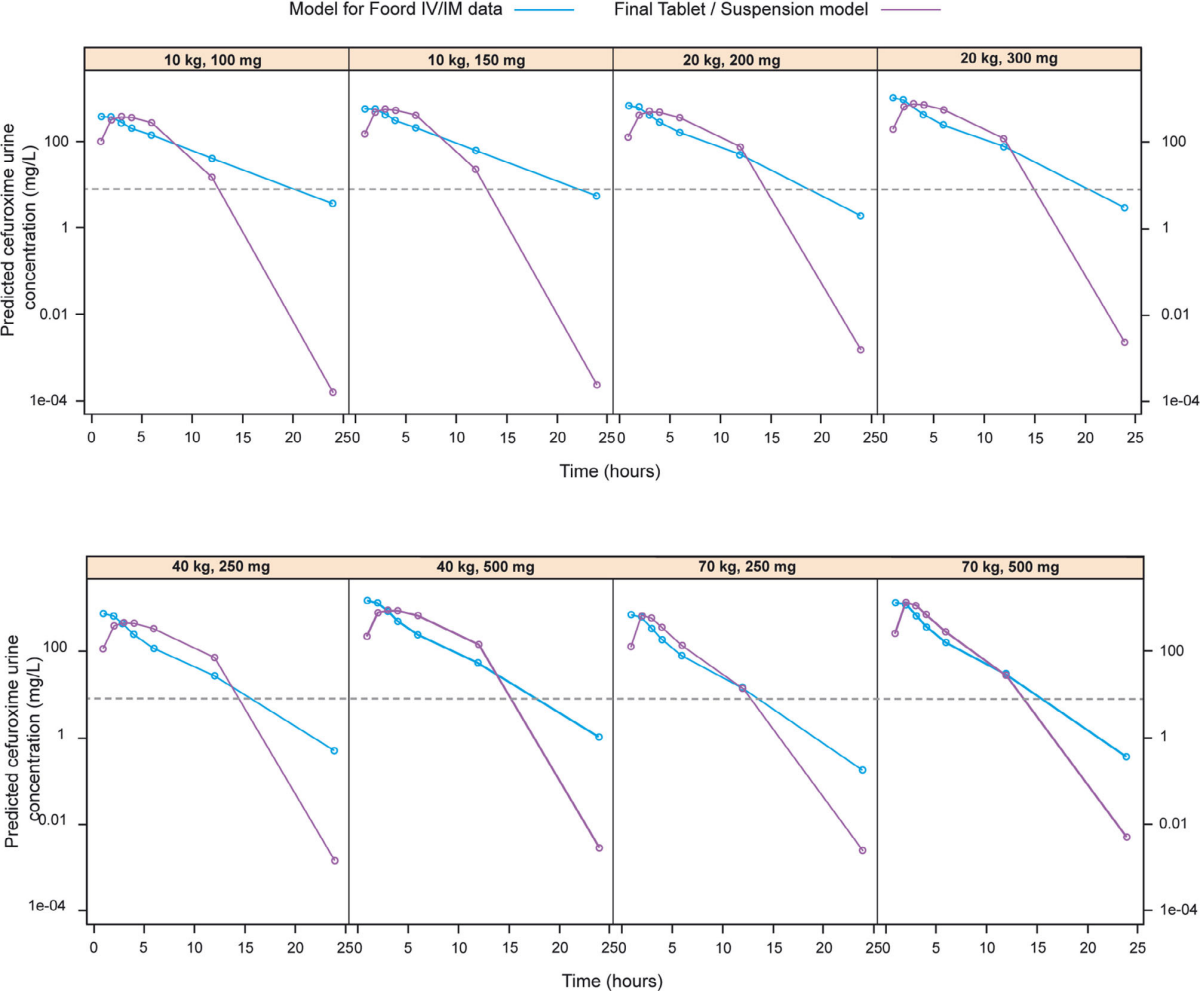
Predicted cefuroxime concentration *vs.* time profiles in urine following administration of cefuroxime axetil doses between 100 and 500 mg (BID) to paediatric and adult patients based on the adjusted IM/IV model and adapted suspension and tablet model. The dotted line denotes the cefuroxime MIC of *E. coli* at 8 mg/L. BID, twice daily; IM, intramuscular; IV, intravenous; MIC, minimum inhibitory concentration.

Simulated exposure (AUC, Cmax) T > MIC and PTA across different weight bands and dose cohorts are summarized in Tables S14, S15 and S16, respectively. As cefuroxime is almost exclusively renally eliminated into urine,^14^ urine volume production in children was inferred from population data for different age groups, namely, 2 mL/kg/h for infants (e.g., 0.02 L/h for a 1‐year‐old, 10 kg), 1.5 mL/kg/h for toddlers and young children (e.g., 0.03 L/h for a 4‐year‐old, 20 kg), 1 mL/kg/h for older children and adolescents (e.g., 0.04 L/h for an 11‐year‐old, 40 kg), and 0.06 L/h for adults.^24^ Given that cefuroxime data in urine was available only from a study in which cefuroxime axetil has been administered intramuscularly and intravenously, two different parameterisations were considered to obtain profiles following oral administration of tablets or suspension. Whilst numerical differences were observed in the predicted cefuroxime concentrations in urine obtained from each model (Table 1), simulated profiles showed comparable disposition patterns (Figure 4).

**TABLE 1:** Predicted cefuroxime concentration over time in urine using two different pharmacokinetic models: (upper panel) intramuscular/intravenous model, adjusting for tablet and suspension bioavailability; (lower panel) adapted version of the previously built pharmacokinetic model describing cefuroxime pharmacokinetics after oral administration.

Weight (kg)	Unit dose	Predicted urine cefuroxime concentrations (mg/L) at different times (h) postdose:							% bioavailable dose recovered over 24 h
		1 h	2 h	3 h	4 h	6 h	12 h	24 h	
70	250‐mg tablet	682.1	604.3	346.2	187.9	81.1	15.1	0.2	100%
70	500‐mg tablet	1364.3	1208.7	692.5	375.8	162.2	30.2	0.4	100%
40	250‐mg suspension (10 mg/kg)*	748.5	665.8	411.0	244.0	119.6	27.8	0.5	100%
40	500‐mg suspension (15 mg/kg)*	1497.0	1331.6	822.0	487.9	239.1	55.7	1.0	100%
20	200‐mg suspension (10 mg/kg)	657.1	606.4	418.0	280.5	162.7	51.3	1.9	100%
20	300‐mg suspension (15 mg/kg)	985.6	909.6	627.0	420.7	244.0	76.9	2.9	100%
10	100‐mg suspension (10 mg/kg)	370.2	360.5	276.0	207.2	140.1	41.1	3.6	99.3%
10	150‐mg suspension (15 mg/kg)	555.3	540.8	414.1	310.8	210.2	61.7	5.5	99.3%

Weight (kg)	Unit dose	Predicted urine cefuroxime concentrations (mg/L) at different times (h) postdose:							% bioavailable dose recovered over 24 h
		1 h	2 h	3 h	4 h	6 h	12 h	24 h	
70	250‐mg tablet	126.3	645.8	586.8	358.1	139.8	14.3	0	100%
70	500‐mg tablet	252.6	1291.7	1173.5	716.2	279.5	28.6	0	100%
40	250‐mg suspension (10 mg/kg)*	110.1	378.7	455.3	439.6	338.8	72.5	0	100%
40	500‐mg suspension (15 mg/kg)*	220.1	757.4	910.7	879.1	677.7	144.9	0	100%
20	200‐mg suspension (10 mg/kg)	123.8	411.1	487.3	467.7	358.4	76.0	0	100%
20	300‐mg suspension (15 mg/kg)	185.7	616.7	731.0	701.6	537.5	114.0	0	100%
10	100‐mg suspension (10 mg/kg)	97.2	312.6	366.4	350.1	266.8	14.8	0	100%
10	150‐mg suspension (15 mg/kg)	145.7	468.9	549.6	525.1	400.2	22.3	0	100%

* 10 and 15 mg/kg doses were capped to a maximum dose of 250 mg and 500 mg, respectively per administration.

The amount excreted in urine was converted to concentrations by dividing it by the urine volume produced at each time point. The model was adjusted to account for the difference in bioavailability between tablets (50%)/suspension (40%) and intramuscular administration (100%).

##### Predicted cefuroxime concentrations in urine using the IM/IV PK model, adjusted for bioavailability

Using the IM/IV model after adjusting for differences in the bioavailability between tablets and suspension, cefuroxime concentrations in urine were simulated over time. Results showed that urine concentrations remain above the required 8 mg/L over the dose interval (T > MIC = 100%) for all simulated weight bands (MIC _susceptibility breakpoint_ for *E. coli*, European Committee on Antimicrobial Susceptibility Testing [EUCAST]^25^, ^26^; Table 1A; Table S16). Based on these predictions, 100% of the patient population can achieve a T > MIC ≥40% (PTA = 100%). The fast absorption rate of IM doses also shows that cefuroxime appears in urine somewhat faster than after oral administration of cefuroxime axetil suspension. Therefore, it is likely that the slower absorption rates of tablets and suspension doses will result in urine concentrations remaining higher for a prolonged time‐period. These differences are unlikely to alter the T > MIC or PTA required for UTI treatment.

##### Predicted cefuroxime concentrations in urine using the PK model for suspension and tablets, adjusted for renal excretion

The extended PK model describing oral absorption and disposition of cefuroxime in serum provided comparable urine excretion profiles to those obtained with the IV/IM PK model (Table S1B). Simulations of the amount of cefuroxime excreted in urine based on compartmental modelling of data from tablets and suspension after oral administration reveal that cefuroxime concentrations in urine remain above the required 8 mg/L over the entire dosing interval after administration of suspension or tablet doses of 10 mg/kg BID or 250 mg BID, respectively, in the relevant population groups. As expected, the slower absorption of tablet and suspension results in a delayed peak concentration in urine relative to that observed following IV/IM dosing; however, this did not affect T > MIC (Figure 4). Given the elimination half‐life of cefuroxime, most of the dose is eliminated within 24 h.

Calculation of the PTA over a relevant range of MICs resulted in similar estimates to those obtained for the IV/IM model. Simulations showed that a T > MIC ≥40% could be maintained across MICs of up to ≥32 mg/L in 100% of simulated patients (Table 1; Table S16).

## 4 DISCUSSION

Changes in bacterial susceptibility can affect treatment efficacy, potentially resulting in clinical failure. Differences in susceptibility occur over different geographical regions and at different rates over time, making it essential that clinicians and prescribers are aware of microbiological susceptibility locally.^27^, ^28^ Such a situation also requires ensuring that the use of old and new antibiotics is optimized for specific pathogens and infections. In addition, it is critical that treatment optimisation is not only based on improving therapeutic outcome but also minimizing the risk of resistance. Even though the efficacy of new antimicrobials is reasonably described, the dose–exposure–response relationships have been updated only for few of the older agents to consider changes in bacterial susceptibility.^6^, ^8^, ^26^ Here we have explored the consequence of such an evolution for cefuroxime and its implications for dosing recommendations using modelling and simulation. Specifically, we have evaluated the implications of intrinsic and extrinsic sources of variability in cefuroxime PK to establish the suitability of various doses and dosing regimens across different indications. This approach has been possible because of the considerable advances made over the past 2 decades, particularly regarding PK and pharmacodynamic principles.^29^, ^30^, ^31^, ^32^, ^33^, ^34^

Our analysis highlights that optimizing dose and dosing regimens requires an understanding of the effect of differences at individual‐patient level (including information on the dose, formulation and dosing regimen), whereas emergence of resistance and changes in susceptibility are an ecologic issue. It is essential that clinical decisions be based on the underlying exposure–response relationships, translated in practical terms as PTA.

### 4.1 PTA and antibacterial activity

This study aimed to address questions regarding the ability of switching between suspension and tablet formulations of cefuroxime axetil in paediatric and adult patients at similar doses, and providing insight into the PTA of the various available doses and dosing regimens considering microbiological susceptibility across a wide MIC range.

While the approach presented here relies on summary‐level data for cefuroxime, the available publications provided the information required to ensure accurate characterization of its PK.^3^, ^14^, ^15^, ^16^ Population PK models were developed that described the overall cefuroxime concentration*–*time profiles observed after administration of different formulations, dose levels and across a wide group of individuals, including paediatric and adult patients. Model‐derived apparent clearance (34.0 L/h) was slightly higher than previously reported data (23.0 L/h).^19^ However, measures of AUC and Cmax showed good agreement between those observed in the individual studies, and those predicted by the model.

Weight is generally considered a better predictor for cefuroxime PK, and indeed our investigation did not reveal an effect of age after taking weight into account. Typically, cefuroxime axetil dose recommendations are provided on the basis of weight. For this reason, our simulations were stratified for weight, but in some cases dose adjustments are given based on age ranges. To facilitate the interpretation of our results across age ranges, the weight ranges in this study could approximately be translated to age ranges as follows: 4–<6 kg (0.25–1 year), 6–<12 kg (1–2 years), 12–<20 kg (2–4 years), 20–<40 kg (4–11 years), 40–<60 kg (>12 years), 60–<80 kg (>16 years) and 80–<100 kg (>16 years).

Comparison of the PTA resulting from tablet and suspension at similar doses showed that T > MIC is maintained over a longer period following administration of the suspension due to its slower absorption rate relative to tablets. Nevertheless, simulation scenarios indicate that switching from tablets to suspension does not lead to a reduction of the PTA below the target of 90% for MICs ≤0.5 mg/L.

Our analysis has also confirmed that the lower doses of cefuroxime axetil (10 mg/kg BID in patients weighing ≤40 kg and 250 mg BID in patients weighing >40 kg) appear to be appropriate for the treatment of infections caused by pathogens with an MIC ≤0.25 mg/L. This lower dose is typically used for infections caused by more susceptible pathogens, where MIC tends to be well below that threshold, for example with infections of *S. pyogenes* (MIC ≤0.25 mg/L). By contrast, higher doses of cefuroxime axetil (15 mg/kg BID in patients weighing ≤40 kg and 500 mg BID in patients weighing >40 kg) were found to be appropriate for the treatment of infections caused by pathogens with an MIC ≤0.5 mg/L. Although EUCAST lists the MIC for *S. pneumoniae* to be ≤0.25 mg/L, the CLSI lists it to be ≤1.0 mg/L. As shown in Figure 1, such susceptibility may vary significantly between regions and across time, requiring the prescriber to make an informed decision on the appropriate dose. Our results show, however, that adequate exposures to achieve a PTA > 90% for MICs ≤0.5 mg/L are likely to be achieved in patients weighing ≤100 kg.

The most common pathogen for UTIs with regard to antibiotic treatment is *E. coli*, with a cefuroxime MIC of 8 mg/L. An assessment of urine cefuroxime concentrations over time based on an extrapolated IM/IV model, as well as an adapted version of the final model for suspension and tablets, showed that concentrations are likely to be above the required 8 mg/L to treat UTIs caused by *E. coli*. An analysis of the PTA for cefuroxime axetil suspension or tablet doses of 10 mg/kg BID or 250 mg BID will probably result in T > MIC≥40% in 100% of the patients. This assessment confirmed that these doses more than adequately cover the key pathogen for UTIs.

Cefuroxime axetil is mostly prescribed empirically, i.e., without conclusive evidence of the causative bacteria or their susceptibility to treatment. Hence, it is important that currently approved doses are shown to cover the most common MICs for the most prevalent causative bacteria. Table S7 provides the MICs of the most common causative bacteria covered by different cefuroxime axetil dosage regimens across the labelled indications. However, local susceptibility and resistance patterns relevant to each case may not be reflected. In fact, there are local guidelines suggesting 1000 mg BID dose for uncomplicated respiratory tract infections in certain regions.^35^, ^36^

### 4.2 Limitations

Although both PK models were fit for purpose and accurately described the concentration*–*time profiles in plasma/serum and urine, there are some limitations to our analysis, which need to be carefully considered if the model and underlying assumptions are to be used subsequently in future endeavours. First, the PK models were built on summary‐level data alone, and required imputation of weight in some places. This led to an inability to perform a thorough analysis of the effect of other potential covariate factors, and it was assumed that the cefuroxime PK adhered to established allometric principles, an assumption that tends to hold well for renally cleared drugs.^20^ In practice, it means that estimates of IIV were primarily determined by interstudy and intercohort variability, probably associated with differences in the study population. However, comparing the mean and standard deviation of simulated and observed AUC and Cmax for the different subgroups showed that the models were able to adequately reproduce means, as well as IIV compared to the literature sources. Due to a lack of individual‐level data, some IIVs may not have been captured fully; however, this is unlikely to affect the PTA, as such IIV becomes less relevant when simulations are performed with large numbers of patients. Furthermore, it was not possible to assess the implications of individual differences in creatinine clearance on cefuroxime exposure, as this was not recorded in the source data and thus not included in the model. All simulations were based on the assumption of a patient population with normal renal function, in whom fluctuations in creatinine clearance were likely to have minor or no effect on total clearance. Although it is known that the PK of antibiotics and antivirals may be affected by severe disease,^12^, ^23^, ^33^ the current data were based only on healthy individuals and patients with relatively mild infections, and thus the predictions for T > MIC and PTA may not translate well to patients with severe disease.

While in an ideal setting simulations would have been performed with models derived from the analysis of individual‐level data, we anticipate that any further extension of these models to incorporate additional intrinsic and extrinsic sources of variability is unlikely to significantly alter the current results and conclusions from our analysis.

Similarly, with regard to the calculation of cefuroxime concentrations in urine, it can be anticipated that the use of average urine formation rates, variable bladder size and consequently voiding volume across the paediatric and adult populations should not affect the conclusions about the PTA for the treatment of UTIs. The observed and predicted amount excreted in urine is such that target exposure is achieved even if IIV in renal clearance is assumed to be high. Moreover, the prediction of urine excretion following oral doses of suspension and tablets has relied on the extrapolation of bioavailability and renal clearance across dosage forms and age groups. These assumptions are unlikely to be invalidated by unaccounted variability, as the predicted urine concentrations exceed the target MIC of 8 mg/L significantly.

### 4.3 Clinical implications

Based on the MICs of the key pathogens for each indication, the dosage regimen can be optimized for some indications (Table S7). While the EUCAST breakpoint for *S. pneumoniae*, the key pathogen for acute otitis media and acute bacterial sinusitis, is 0.25 mg/L, most of its isolates have an MIC >0.25 mg/L (Figure 1). An increase of the adult dose of 250–500 mg BID could improve the PTA for these indications, to account for these isolates. Likewise, a dose of 500 mg BID may be required to cover *Staphylococcus aureus* isolates with high MIC involved in some skin and soft tissue infections. Our results also showed that the current dose of 250 mg BID should suffice to cover the key pathogen of *E. coli* in adult UTIs. However, as adults with UTIs may often encounter more resistant variants, the higher dose of 500 mg BID could be used in cases of suspected reduced susceptibility.

Similar to adults, a 250 mg BID tablet dose should suffice for acute tonsillitis or pharyngitis in children weighing 20–40 kg. For all other indications, however, the higher tablet dose of 500 mg BID may be necessary. For the suspension formulation, the dose of 10 mg/kg BID capped at 500 mg/day for acute tonsillitis and pharyngitis, and 15 mg/kg BID capped at 1000 mg/day for all other indications should provide sufficient coverage.

It is important to note that our analysis does not preclude the possibility of local differences in susceptibility for the relevant pathogens, and as the cefuroxime axetil label specifies, physicians should consider local guidelines and susceptibility trends.

## 5 CONCLUSION

Our analysis allowed the characterization of cefuroxime PK after administration of different formulations and dosing regimens of cefuroxime axetil to adults and children, showing that switching between tablet and suspension should not affect efficacy. We also provided extensive evidence that may enable prescribers to optimize cefuroxime axetil doses across indications and populations based on the susceptibility profiles. Further modelling and simulation studies may be required to assess the potential clinical consequences of variation in bacterial susceptibility between countries or regions on the ability to achieve therapeutic targets locally.

## AUTHOR CONTRIBUTIONS

O.D.P. and S.C.v.D. designed the investigation protocol, S.C.v.D. performed the analysis. S.C.v.D., P.K., J.K. and O.D.P. contributed to interpretation of the data, writing the manuscript and approved its submission for publication.

## CONFLICT OF INTEREST STATEMENT

S.C.v.D., J.K., P.K. and O.D.P. are employees of GSK, and S.C.v.D., P.K. and O.D.P. are shareholders in GSK.

## Supporting information


**FIGURE S1** Diagram of the steps involved in data selection, model building, model validation, clinical trial simulations and prediction of cefuroxime concentrations (systemic and in urine) along with the corresponding PTA for the different dosage forms and dosing regimens. CFM, cefuroxime; CTS, clinical trial simulations; IM, intramuscular; IV, intravenous; VPC, visual predictive check
**TABLE S1** (A) Doses, dosing regimens and demographic characteristics of the study populations used for the analyses. (B) Sample matrix and drug concentrations reported in the studies included in the analyses.
**TABLE S2** Overview of the imputations of weight per data source and cohort
**FIGURE S2** Comparison between cefuroxime observed concentrations (blue circles), and predictions using the regular first‐order absorption kinetics (red line) and time‐dependent absorption kinetics (green line) after a single dose of 250‐mg cefuroxime axetil suspension. KA, absorption rate constant (h^‐1^).
**TABLE S3** Parameter values and their precision (RSE%) for the interim and final models, including shrinkage estimates.
**FIGURE S3**. Goodness‐of‐fit plots for the interim model following oral administration of tablet, crushed tablet and suspension to adults and children. Open circles show individual data points. Solid red line is a trend line. CWRES, conditional weighted residuals
**FIGURE S4** Visual predictive check for the interim model, stratified by formulation. Blue circles and lines: observed concentrations and median; black solid and dotted lines: median and 90% prediction intervals.
**FIGURE S5** Model performance assessed by visual predictive check of the external validation data from Powell *et al*.^3^ using the interim oral administration model, stratified by dose. Data only in infants and children. Blue circles and lines: observed concentrations and median; black solid and dotted lines: median and 90% prediction intervals
**FIGURE S6** Goodness‐of‐fit plot for the final model following oral administration of tablet, crushed tablet and suspension to adults and children. Open circles show individual data points. Solid red line is a trend line. CWRES, conditional weighted residuals.
**FIGURE S7** Visual predictive check for the final model following oral administration of tablet, crushed tablet and suspension to adults and children, stratified by formulation. Blue circles and lines: observed concentrations and median; black solid and dotted lines: median and 90% prediction intervals.
**FIGURE S8** Density plots of the eta distributions for CL, Vd and Ka from the final model following oral administration of tablet, crushed tablet and suspension to adults and children. CL, clearance; Eta, NONMEM interindividual variability; Ka, absorption rate constant; Vd, volume of distribution.
**FIGURE S9** Observed (open circles) and predicted cefuroxime concentrations in plasma or serum stratified by study arm, and population (adult *vs.* children). Predicted cefuroxime concentrations simulated using the final model following oral administration of tablet, crushed tablet and suspension to adults and children. T, tablet; C, crushed tablet; S, suspension.
**TABLE S4** Reported and model‐predicted mean estimates of AUC_0‐∞_ and Cmax for cefuroxime along with the corresponding ratio between observed and predicted parameters.
**FIGURE S10** Diagram of the model structure describing the PK of cefuroxime after IM/IV administration of cefuroxime axetil. CFX, cefuroxime; CL, clearance; IM, intramuscular; IV, intravenous.
**TABLE S5** Parameter estimates along with their precision (RSE%) and shrinkage for the IM/IV model.
**FIGURE S11** Observed and predicted cefuroxime concentrations in serum after IM or IV administration. Results are shown stratified by route of administration and dose level (treatment arm). IM, intramuscular; IV, intravenous.
**FIGURE S12** Observed and predicted cefuroxime amount in urine after IM or IV administration. Results are shown stratified by route of administration and dose level. IM, intramuscular; IV, intravenous.
**FIGURE S13** Predicted concentration*–*time profile following cefuroxime doses of 10 mg/kg BID for ≤40 kg and 250 mg BID for >40 kg, stratified by weight bands. Median AUC and time above MIC (T > MIC) along with the 95% confidence interval are summarized for MIC = 0.25 mg/mL. Curves depict median profiles and corresponding 95% prediction interval.
**FIGURE S14** Predicted concentration–time profile following cefuroxime doses of 15 mg/kg BID for ≤40 kg and 500 mg BID for >40 kg, stratified by weight bands. Median AUC and time above MIC (T > MIC) along with the 95% confidence interval are summarized for MIC = 0.25 mg/mL. Curves depict median profiles and corresponding 95% prediction interval.
**TABLE S6** Dose conversion from suspension to tablets in patients with body weight 20–40 kg, who are able to swallow tablets of 250 mg.
**TABLE S7** Cefuroxime axetil indications, common causative pathogens, CLSI and EUCAST MIC susceptibility breakpoints.
**TABLE S8** AUC and Cmax in plasma following administration of lower (10 mg/kg) and higher (15 mg/kg) doses of cefuroxime, as implemented in simulation scenario 1. Values are mean (SD).
**TABLE S9** T > MIC based on plasma cefuroxime concentrations, as implemented in simulation scenario 1. Values are mean (SD).
**TABLE S10** Probability of target attainment based on plasma cefuroxime concentrations, as implemented for simulation scenario 1.
**TABLE S11** AUC and Cmax in plasma, as implemented in simulation scenario 2. Values are mean (SD).
**TABLE S12** T > MIC based on plasma cefuroxime concentrations, as implemented in simulation scenario 2. Values are mean (SD).
**TABLE S13** Probability of target attainment based on plasma cefuroxime concentrations, as implemented for simulation scenario 2.
**TABLE S14** AUC and Cmax in urine, as implemented in simulation scenario 3. Values are mean (SD).
**TABLE S15** T > MIC based on urine cefuroxime concentrations, as implemented in simulation scenario 3. Values are mean (SD).
**TABLE S16** Probability of target attainment based on urine cefuroxime concentrations, as implemented for simulation scenario 3.

## Data Availability

The data that support the findings of this study are available from the corresponding author upon reasonable request.
