# Supplementary material for: Cefuroxime axetil dosing regimens and probability of target attainment in adults and children
Source: Br J Clin Pharmacol. 2025 Jul 17;91(11):3213–24. doi: 10.1002/bcp.70158 (PMC12569549; doi:10.1002/bcp.70158)
Supplement: Supplementary file 1 — FIGURE S1 Diagram of the steps involved in data selection, model building, model validation, clinical trial simulations and prediction of cefuroxime concentrations (systemic and in urine) along with the corresponding PTA for the different dosage forms and dosing regimens. CFM, cefuroxime; CTS, clinical trial simulations; IM, intramuscular; IV, intravenous; VPC, visual predictive check TABLE S1 (A) Doses, dosing regimens and demographic characteristics of the study populations used for the analyses. (B) Sample matrix and drug concentrations reported in the studies included in the analyses. TABLE S2 Overview of the imputations of weight per data source and cohort FIGURE S2 Comparison between cefuroxime observed concentrations (blue circles), and predictions using the regular first‐order absorption kinetics (red line) and time‐dependent absorption kinetics (green line) after a single dose of 250‐mg cefuroxime axetil suspension. KA, absorption rate constant (h‐1). TABLE S3 Parameter values and their precision (RSE%) for the interim and final models, including shrinkage estimates. FIGURE S3. Goodness‐of‐fit plots for the interim model following oral administration of tablet, crushed tablet and suspension to adults and children. Open circles show individual data points. Solid red line is a trend line. CWRES, conditional weighted residuals FIGURE S4 Visual predictive check for the interim model, stratified by formulation. Blue circles and lines: observed concentrations and median; black solid and dotted lines: median and 90% prediction intervals. FIGURE S5 Model performance assessed by visual predictive check of the external validation data from Powell et al.3 using the interim oral administration model, stratified by dose. Data only in infants and children. Blue circles and lines: observed concentrations and median; black solid and dotted lines: median and 90% prediction intervals FIGURE S6 Goodness‐of‐fit plot for the final model following oral administration of tablet, [file BCP-91-3213-s001.pdf]

SUPPLEMENTARY MATERIAL

1. Introduction and high-level summary

This appendix details the methods and results for model building and the simulations of cefuroxime concentrations (systemic and in urine). A diagram of the modelling steps can be seen in **Fig. S1**. In short, data on cefuroxime concentrations in plasma or serum and urine was collected from four literature sources, converted into a standard NONMEM data set format, with imputation of weight and age based on certain assumptions (see Section 2). Subsequently, standard model-building methods using a nonlinear mixed effects approach were applied, where relevant, to characterise the correlations between cefuroxime axetil dose and its systemic and urine concentrations. Due to large differences in the pharmacokinetic (PK) data between the parenteral and enteral administration of the cefuroxime, separate models were built, one characterising systemic cefuroxime PK after administration of tablets, crushed tablets and suspension, and another model characterising systemic and urine cefuroxime PK after intramuscular (IM) and intravenous (IV) administration. For the oral administration model, an interim model was built on a subset of data from two studies. Subsequently, the interim model was used to predict systemic cefuroxime concentrations in a third study. Afterwards, the model was refit to the full data set from the three studies involving oral administration (final model). Using the final model, clinical trial simulations were performed to assess the time above minimum inhibitory concentration (T>MIC) and probability of target attainment for various dosing and formulation scenarios. On the other hand, the model describing the IM/IV data was developed in a single step, as serum and urine data from Foord, 1976 [4] was insufficient to perform internal and external validation through data splitting or bootstrapping. The IM/IV model showed that the entire delivered amount of cefuroxime can be considered renally eliminated. This allowed the adaptation of the oral dose model to describe cefuroxime concentrations in urine. We provide predictions of urine concentrations from both models for comparison.

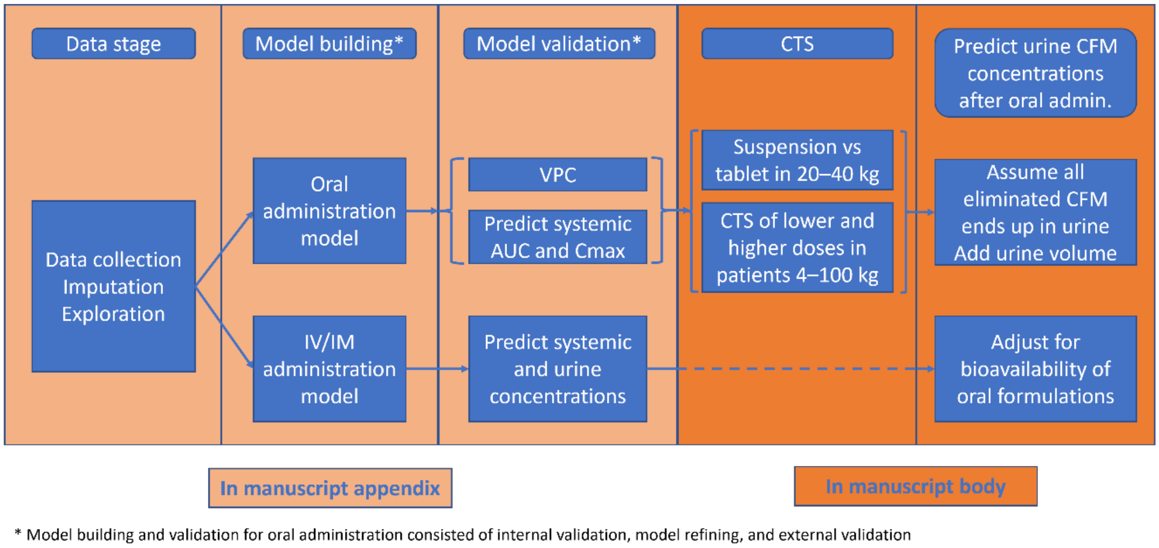

Figure S1. Diagram of the steps involved in data selection, model building, model validation, clinical trial simulations, and prediction of cefuroxime concentrations (systemic and in urine) along with the corresponding PTA for the different dosage forms and dosing regimens.

CFM, cefuroxime; CTS, clinical trial simulations; IM, intramuscular; IV, intravenous; VPC, visual predictive check.

2. Data available for model building

Table S1 lists the different sources from which the modelling data set was derived. From a total pool of 139 individuals in the four studies listed in **Table S1**, 95 had accurate details regarding the weight range and 71 had mean estimates for age. The majority of the patients (*n*=102) were male (73.4%), 61 were paediatric patients (43.9%) and 78 were adults (56.1%). Of the total pool, 28 individuals received the suspension, whilst 43 individuals were administered the tablets and 24 individuals received both suspension and tablet in a crossover design. In addition, data from 44 adult individuals receiving IV and IM doses of cefuroxime were used as reference for urine excretion. Out of a total of 139 individuals, 78 were healthy volunteers and 61 were patients with various infections.

In total, summary level PK data relative to 67 individuals reported by Ginsburg et al. 1985 [1] and Donn et al. 1994 [2] were used for model building. This subset included paediatric

patients and adult healthy individuals receiving an oral dose of cefuroxime axetil tablet or suspension. While the crushing of cefuroxime tablets is not recommended in the current label, it was investigated in the Ginsburg study. Subsequent steps for model validation and refinement were based on data from 28 paediatric patients receiving oral doses of the suspension [3]. In addition, during model building, it became evident that the development of an integrated model including both systemic and urine concentrations could not be supported by the available data. Therefore, the data from Foord, 1976 [4] was excluded from the primary model building and analysed separately using compartmental methods.

Individual participant weight was not reported in any of the data sources. Consequently, mean weights were used for each study cohort, where available. For Foord, 1976 [4] and Donn, 1994 [2], weights of 70 kg were imputed. For Powel, 1991 [3], the mean weight of each dose cohort was used. For the adult cohort in Ginsburg, 1985 [1], a mean weight of 69.5 was used. For the younger and older children cohorts in Ginsburg, 1985 [1], weight was imputed based on the average age of each cohort, using the formula weight (kg) = age (years) * 3 + 7 [5]. **Table S2** shows the weights used for each cohort from each data source. Given this direct relation between weight and age in our data, age was not included as a covariate column in the data.

Table S1.A Doses, dosing regimens and demographic characteristics of the study populations used for the analyses.

Demographic	Foord, 1976[4]	Ginsburg, 1985[1]			Powell, 1991[3]	Donn, 1994[2]	Overall
Population	Adults	Younger children	Older children	Adults	Infants and children	Adults	Infants, children and adults
Indication	Healthy individuals	Acute otitis media or skin and soft-tissue infections		Healthy individuals	Facial cellulitis (16)<br>Pneumonia (11)<br>Cervical adenitis (1)	Healthy individuals	Healthy individuals and patients
Number of individuals	44	22	11	10	28	24	139
Age (years)<br>Mean [range]	-<br>[19–57]	2.8<br>[0.9–5.7]	11.1<br>[7.6–12.3]	27<br>[24–31]	1.9<br>[0.25–12]	-<br>[19–38]	-<br>[0.25–57]
Weight (kg)<br>Mean [range]	-<br>[-]	22.8<br>[9.1–51.2]		69.5<br>[51–94]	11.1<br>[5–47]	-<br>[62–84]	-<br>[5–94]
Sex (M:F)	44:0	18:15		5:5	11:17	24:0	102:37
Formulations and dose administered	IV bolus:<br>250 mg<br>500 mg<br>1000 mg<br>IM:<br>250 mg<br>500 mg<br>750 mg<br>1000 mg	Crushed tablets:<br>15 mg/kg (F)<br>15 mg/kg (M)<br>20 mg/kg (F)<br>20 mg/kg (M)	Tablets:<br>500 mg (F)<br>500 mg (M)<br>500 mg (A)	Tablets:<br>500 mg (F)<br>500 mg (M)<br>500 mg (A)	Suspension:<br>10 mg/kg (M)<br>15 mg/kg (M)<br>20 mg/kg (M)	Tablet:<br>250 mg ("Fed")<br>Suspension:<br>250 mg ("Fed")	IV, IM and oral administration.<br>Solution suspension and tablets

Age and weight given as mean [range]. (F): given when fasting, (M): given after milk, (A): given after apple sauce, (Fed): given after a standard breakfast. In Foord, 1976 [4], Ginsburg et al. 1985 [1] and Powell et al. 1991 [3], cefuroxime concentrations were determined after a single dose. In Donn et al. 1994 [2], cefuroxime concentrations were determined at steady state.

IM, intramuscular; IV, intravenous.

Table S1.B Sample matrix and drug concentrations reported in the studies included in the analyses.

	Foord, 1976*[4]	Ginsburg, 1985 [1]	Powell, 1991 [3]	Donn, 1994 [2]	Overall
Data used for model building	serum concentrations and urine amounts	plasma concentrations	serum concentrations	serum concentrations	concentrations in serum, plasma and urine
Systemic cefuroxime concentration mg/L mean (SD)	16.54 (19.51)	2.90 (1.72)	1.33 (0.93)	3.35 (1.37)	7.20 (13.13)
Systemic cefuroxime concentration mg/L median [range]	10.85 [0.30–99.2]	2.80 [0.17–7.4]	1.50 [0.01–3.1]	3.52 [0.92–5.64]	2.80 [0.01–99.2]
Urine cefuroxime concentration mg/L mean (SD)	87.18 (104.61)	-	-	-	87.18 (104.61)
Urine cefuroxime concentration mg/L median [range]	44.25 [1–536]	-	-	-	44.25 [1–536]

*Urine concentrations derived from Foord, 1976 were obtained by taking amounts and dividing them by the mean urine volume produced over the same time period.

IM, intramuscular; IV, intravenous.

Table S2. Overview of the imputations of weight per data source and cohort.

Data source	Cohort name	Age mean (years)	Mean weight (kg)
Foord, 1976 [4]	Adults	>18	70*
Ginsburg, 1985 [1]	Adults	>18	69.5
	Infants and children	2.75	15.3*
	Older children	11.1	40.3*
Powell, 1991 [3]	10 mg/kg	1.51	9.8
	15 mg/kg	1.75	10.1
	20 mg/kg	2.47	13.9
Donn, 1994 [2]	Adults	>18	70*

*Imputed mean weight

3. Model building

3.1. General model-building considerations

Both one- and two-compartmental models were evaluated. Absorption kinetics was parameterised using the absorption rate constant (Ka) and bioavailability (F1). Different absorption models were tested, including first-order time-invariant, semi-physiological [6] and Weibull-type absorption [7]. Given the lack of individual patient-level data, all volume and clearance parameters (CL, V1, Q, V2) were allometrically scaled a priori (allometric exponent of 1 for volumes and 0.75 for clearance and intercompartmental clearance). This assumption offers a pragmatic, robust solution to overcome the lack of individual patient-level data, as the effect of body weight on disposition parameters has been described for a wide range of compounds showing both hepatic and/or renal elimination [8–10]. In many cases, differences in body weight are the primary explanatory factor for interindividual variation in exposure. Given the limited number of infants and very young children (i.e., < 1 year old) in the available studies, it was not possible to discriminate the effect of developmental growth (i.e., allometry) from the potential contribution of maturation processes in renal function to the changes in drug disposition.

A first-order absorption rate process was adequate to describe the general absorption curve for tablets but not the less steep profiles (i.e. slower absorption) of the suspension. The absorption process was therefore assumed to vary over time and described by a time-dependent function:

$$Ka = Ka_{base} * (1 - e^{-1*(K*Time)^{\gamma}})$$

where Ka is the absorption rate constant, Ka_{base} the baseline or initial absorption rate constant value, K is the rate at which Ka increases over time, and γ is the slope or steepness of relationship.

Each of the aforementioned parameters (Ka_{base}, K, γ) as well as the oral bioavailability (F1) were estimated separately for each formulation. Tablets, crushed tablets and suspension were each treated as different formulations. In addition, the value of F1 for tablets was fixed to 1 (100%). As a result, the F1 for crushed tablet and suspension were estimated as relative to that of the tablet. It is important to note that whilst estimates of relative bioavailability in this context are necessary to ensure appropriate description of the concentration vs. time profile for each formulation and mode of intake (i.e. with food, milk, apple sauce), they are apparent values and therefore do not represent the actual bioavailability of the different dosage forms. Due to model instability, data from fasted states (FOOD=0 in the data) were excluded from the analysis. This decision was deemed appropriate for the purposes of the current analysis, as dosing guidelines recommend taking cefuroxime after a meal.

Variability components on population PK parameters were log-normally distributed as a standard assumption for PK parameter distributions. Given that summary data level was used during the parameter estimation steps, interindividual variability as described by the final model also reflected differences between dose levels and studies, which cannot be fully disentangled without PK data in individuals. The variance parameter for Ka was shared between each of the formulations, as there was insufficient data to estimate it separately for each formulation. Residual variance was characterised initially by both proportional and additive components; if either of these were estimated to be close to zero or could not be estimated with reasonable precision (<40% relative standard error [RSE]), it was removed from the model.

Graphical and statistical criteria along with consideration of the underlying scientific rationale was used to identify which covariates should be included or evaluated as influential factors on PK parameters. Given the nature and objective of this analysis, covariate model building was limited to demographic, clinical and other relevant factors known to alter the PK of cefuroxime

(e.g. CL, Ka). The available covariates were body weight (kg), age (years) and treatment conditions (fed and fasting). None of study protocols in which the PK of cefuroxime axetil was evaluated in patients suggested clinical conditions that could lead to disease-related changes in drug disposition, such as critical disease [11].

Allometric correlations between body weight and disposition parameters were fixed a priori. Subsequent covariate model building was based on a stepwise forward addition-backward elimination procedure. The likelihood ratio test was used to evaluate the significance of incorporating or removing fixed effects into the population model based on alpha levels that are set a priori. For forward and backward selections, a significance level of 0.01 for FOCE-I was used. Each covariate individually was included in the base model to identify significant covariates where significance is a reduction in the objective function value of ≥3.84, $\chi^2 < 0.05$ for 1 degree of freedom using FOCE-I. No further covariates were identified after the a-priori inclusion of allometry, and differentiation of absorption kinetic parameters between suspension and tablet formulations. Thus, no backwards elimination of covariates was undertaken.

Initially, model performance was evaluated in terms of parameter precision and bias. RSE% obtained from the covariance step in NONMEM was used to assess the precision of parameter estimates and robustness of the model obtained with the model building data set. Considering the sparse nature of the data, no hard limits were set to this RSE%, but a value of 40% or higher for any parameter was considered reason for further development of the model.

Visual predictive checks (VPC) were used to assess the adequacy of the parameter estimates of models, including their variance, and the effect of covariates. In the VPC, 1000 replicates of the original data set were simulated, based on the final model obtained with each data set along with the 90% prediction intervals. The observed cefuroxime concentrations were plotted over time along with the prediction intervals to visually assess the concordance between simulated and observed data. The interim model built on data from Ginsburg et al. 1985 [1] and Donn et al. 1994 [2] was externally evaluated using a VPC to describe each of the three dose arms in Powell et al. 1991 [3], without re-estimation of the initial population parameters. Finally, model parameters were re-estimated to reflect the combined data from Ginsburg et al. 1985 [1], Powell et al. 1991 [3] and Donn et al. 1994 [2]. This step was considered to yield the final model. In addition to VPCs, model-predicted secondary PK parameters (peak concentration [Cmax] and area under the concentration vs. time curve [AUC]) were compared with mean and standard deviation (SD) reported by Ginsburg et al. 1985 [1], Donn et al. 1994 [2] and Powell

et al. 1991 [3]. Model predictions were based on 1000 replicates of individuals with the same demographics and dose regimen as the original data. Model diagnostic criteria were applied throughout the analysis to ensure appropriate steps were taken to minimize or eliminate bias (i.e. over- or underprediction).

Due to difficulties in consolidating both enteral and parenteral formulations into a single PK model, two separate models had to be implemented. One model described systemic cefuroxime concentrations after administration of tablets, crushed tablets and suspension doses. The second model described systemic and urine cefuroxime concentrations after IM and IV doses. All modelling methods as described above were applied to both models separately, where relevant. Once these models were built, adaptations were made to predict for urine concentrations after administration of tablets or suspension doses. The oral administration model was adapted by assuming all cefuroxime is directly eliminated into urine, and an additional urine volume compartment was added. Model-predicted cefuroxime amounts in urine were converted to urine cefuroxime concentration by dividing the drug amounts by the typical adult volume of urine produced per hour (i.e. 0.06 L/h), with urine volume (in litres) described by the urine compartment. For subsequent steps, urine volume production in children was set at 2 mL/kg/h for infants (e.g. 0.02 L/h for a 1-year-old, 10 kg), 1.5 mL/kg/h for toddlers and young children (e.g. 0.03 L/h for a 4-years-old, 20 kg) and 1 mL/kg/h for older children and adolescents (e.g. 0.04 L/h for an 11-years-old, 40 kg) [8].

To enable comparison of predicted urine concentrations after IV/IM and oral administration, the bioavailability of cefuroxime in the IM/IV model (originally assumed to be 100%) was set to that of the tablet/suspension as described in literature (approximately 50%) [12]. Whilst this assumption does not allow for an accurate description of the slope of drug concentrations in the absorption phase, it ensures accurate description of the data in terms of mass balance, and approximate exposure ranges, serving as a validation step for the description of urinary excretion process following oral administration of cefuroxime.

3.2. Oral administration model

A one-compartment model with Weibull-type absorption was used to describe cefuroxime concentrations over time for each of the dosing regimens (i.e. tablets, crushed tablets, suspension), taking into account the effect of differences in the bioavailability due to the dosage forms. The use of a Weibull function allowed a better description of the protracted absorption

profile seen with the suspension formulation (**Fig. S2**). Parameters were estimated with acceptable precision, with the exception of volume of distribution (**Table S3**). Despite the RSE estimates for the volume of distribution, a sensitivity analysis showed that limited precision in this parameter had no significant effect on the overall estimates of the exposure to cefuroxime, and in particular on the concentration vs. time profile, which are required to calculate the time above minimum inhibitory concentration (T>MIC). Goodness-of-fit plots did not show any bias when comparing observed concentrations against population or individual predictions (**Fig. S3**). No bias was found when comparing the model predictions vs. observations between plasma and serum concentrations. For the purpose of further simulations, and comparison against results from the IM/IV model, no differentiation was made between serum and plasma concentrations.

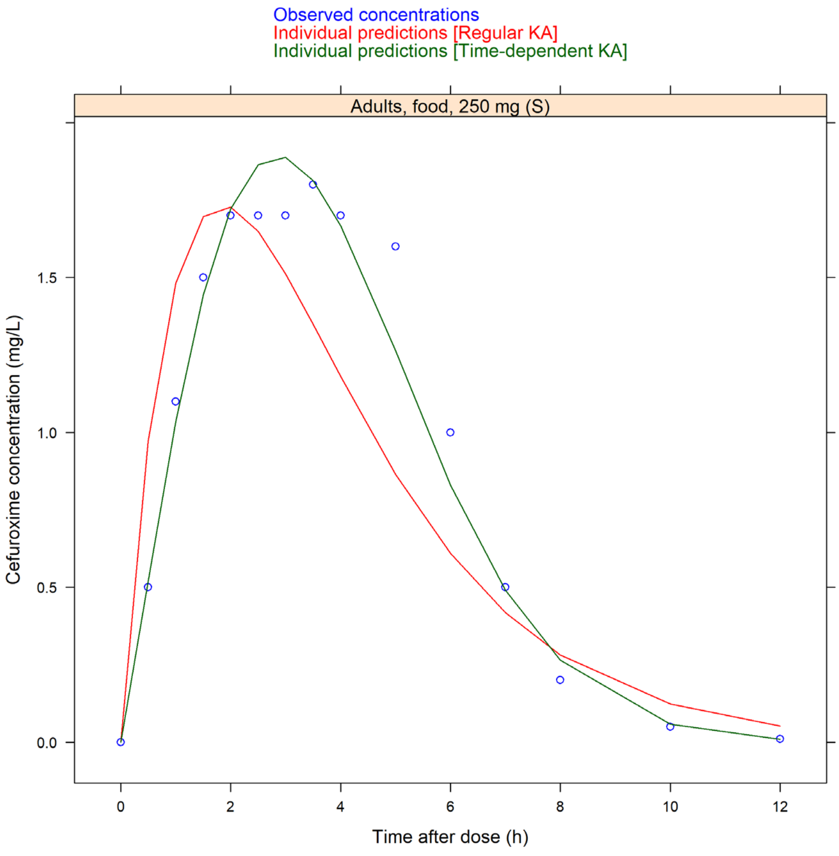

Figure S2. Comparison between cefuroxime observed concentrations (blue circles), and predictions using the regular first-order absorption kinetics (red line) and time-dependent absorption kinetics (green line) after a single dose of 250 mg cefuroxime axetil suspension.

KA, absorption rate constant (h^{-1}). S: suspension

Although the model was built on summary level data, variance estimates were within a reasonable range and similar to that observed in a model built on individual level data [6]. The VPC plots showed that the model was able to describe the profiles associated with different formulations and populations with adequate precision (**Fig. S4**; **Table S3**).

Using the interim model built on the data from Ginsburg et al. 1985 [1] and Donn et al. 1994 [2], model performance was evaluated by predicting the findings reported by Powell et al. 1991 [3]. These results are provided as a VPC (**Fig. S5**). Given the use of summary level data and imputation of covariates to a single value in adult individuals, model performance can be deemed acceptable, despite some minor overprediction. Thus, as a final step, the model was refitted including these data, the results of which are reported in the following section.

Refitting the model to all data from Ginsburg et al. 1985 [1], Powell et al. 1991 [3] and Donn et al. 1994 [2] resulted in a significantly improved precision for volume of distribution and its variance parameter, at the cost of some increase in RSE% for Ka and K for the suspension formulation (**Table S3**). Other parameters showed minor fluctuation relative to the estimates obtained by the interim model. Indeed, the goodness-of-fit plots **(Fig. S6**) showed that the final model describes the data well. In addition, the VPCs (**Fig. S7**) indicate that the final model predicts the overall cefuroxime plasma and serum concentration vs. time profiles for all dosage forms and no longer shows overpredictions for the data reported by Powell et al. 1991 [3]. Furthermore, **Fig. S8** shows no significant bias in the variance of Ka, CL and Vd, although the limited number of individuals results in eta distributions that are not normally distributed. Finally, **Fig. S9** shows that the final model has good prediction performance for each data source.

Variability, as characterised by the final model, showed good agreement between observed and predicted Cmax and AUCs (**Table S4**) for individuals receiving cefuroxime after ingesting food, milk or apple sauce. A notable exception is the 250 mg tablet arm in Donn et al. 1994 [2], for which systemic Cmax and AUC were similar to the 500 mg tablet arm in Ginsburg et al. 1985 [1], possibly pointing to some unknown factors increasing exposure in that particular treatment arm.

Table S3. Parameter values and their precision (RSE%) for the interim and final models, including shrinkage estimates.

Parameter	Interim value (RSE%) [shrinkage %]			Final model value (RSE%) [shrinkage %]		
CL (L/h)	33.1	(11)		34.0	(13)	
Vd (L)	3.79	(168)		7.93	(36)	
Ka (h^{-1}) tablets	0.731	(11)		0.724	(9)	
F1 tablets	1.0	(fixed)		1.0	(fixed)	
K tablets	0.483	(6)		0.523	(5)	
γ tablets	1.66	(4)		1.66	(7)	
Ka (h-1) crushed	0.679	(1)		0.674	(1)	
F1 crushed tablets	0.908	(15)		0.901	(16)	
K crushed tablets	0.574	(8)		0.608	(9)	
γ crushed tablets	1.43	(3)		1.41	(3)	
Ka (h^{-1}) suspension	1.29	(4)		1.76	(48)	
F1 suspension	1.38	(11)		1.26	(14)	
K suspension	0.109	(3)		0.0483	(75)	
γ suspension	1.12	(4)		0.92	(13)	
Ω_{CL}	0.0573	(26)	[0]	0.0472	(23)	[0]
Ω_{Vd}	0.378	(235)	[55]	0.399	(97)	[34]
Ω_{Ka}	0.0102	(32)	[4]	0.0193	(35)	[3]
Σprop	0.0232	(14)	[11]	0.0196	(17)	[14]

CL, clearance; F1, bioavailability; K, rate at which Ka increases over time; Ka, absorption rate constant; RSE, relative standard error; Vd, volume of distribution; γ, Hill coefficient for the rate at which Ka increases over time; Ω_x, variance of parameter *x*; Σprop, proportional residual error.

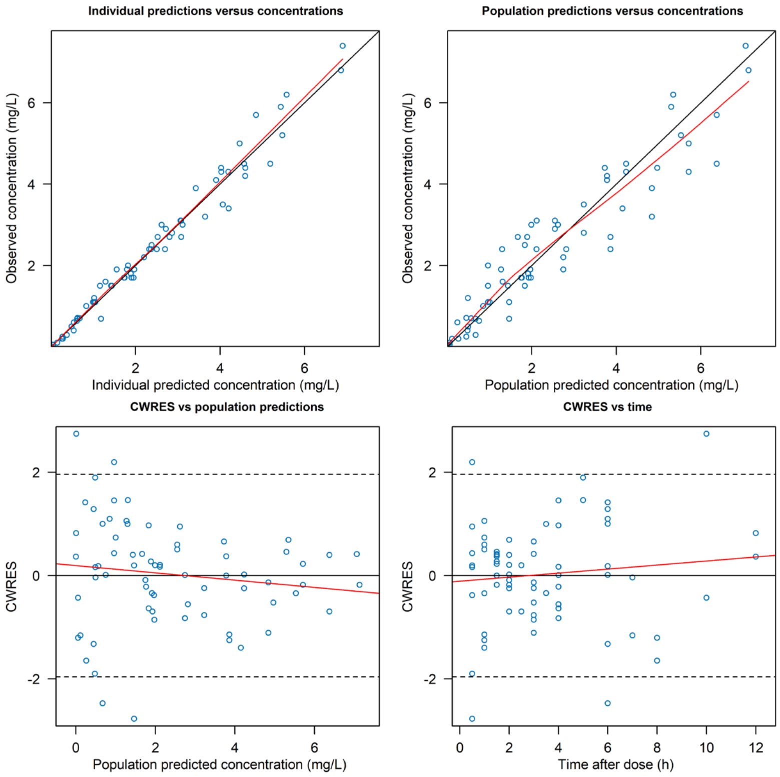

Figure S3. Goodness-of-fit plots for the interim model following oral administration of tablet, crushed tablet and suspension to adults and children.

Open circles show individual data points. Solid red line is a trend line.

CWRES, conditional weighted residuals.

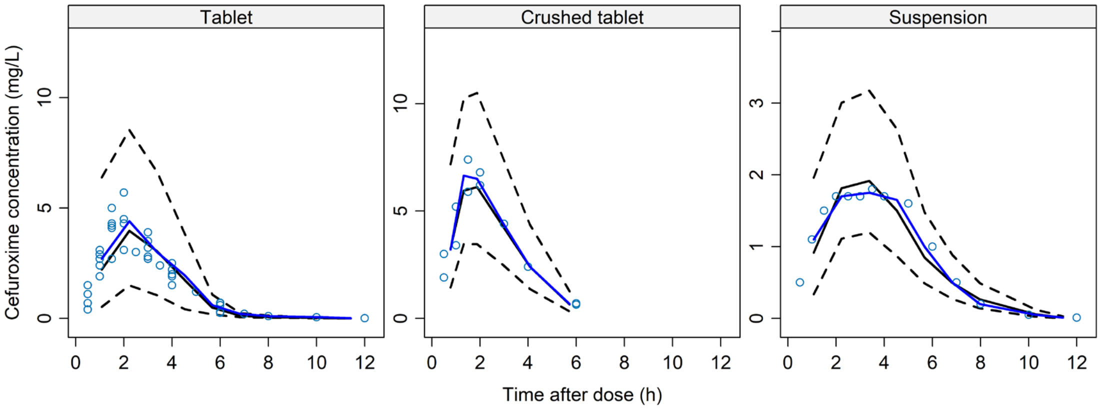

Figure S4. Visual predictive check for the interim model, stratified by formulation.

Blue circles and lines: observed concentrations and median; black solid and dotted lines: median and 90% prediction intervals.

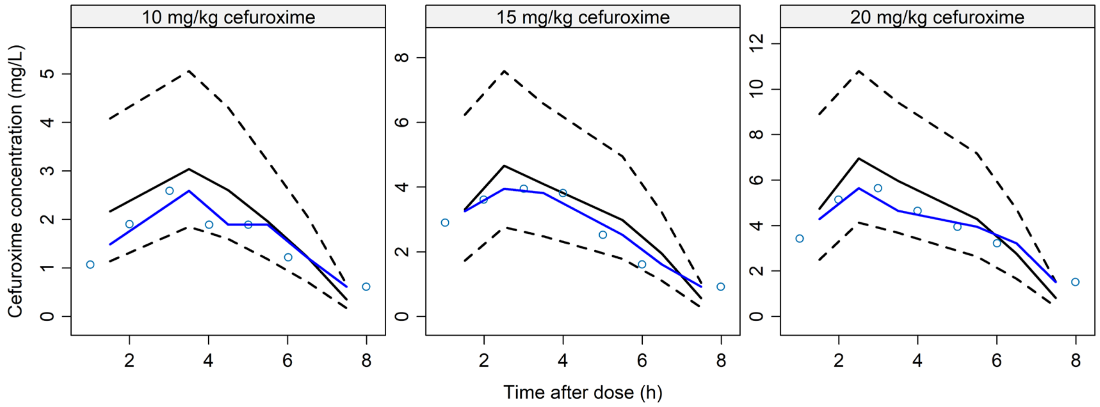

Figure S5. Model performance assessed by visual predictive check of the external validation data from Powell et al. 1991 [3] using the interim oral administration model, stratified by dose.

Data only in infants and children. Blue circles and lines: observed concentrations and median; black solid and dotted lines: median and 90% prediction intervals.

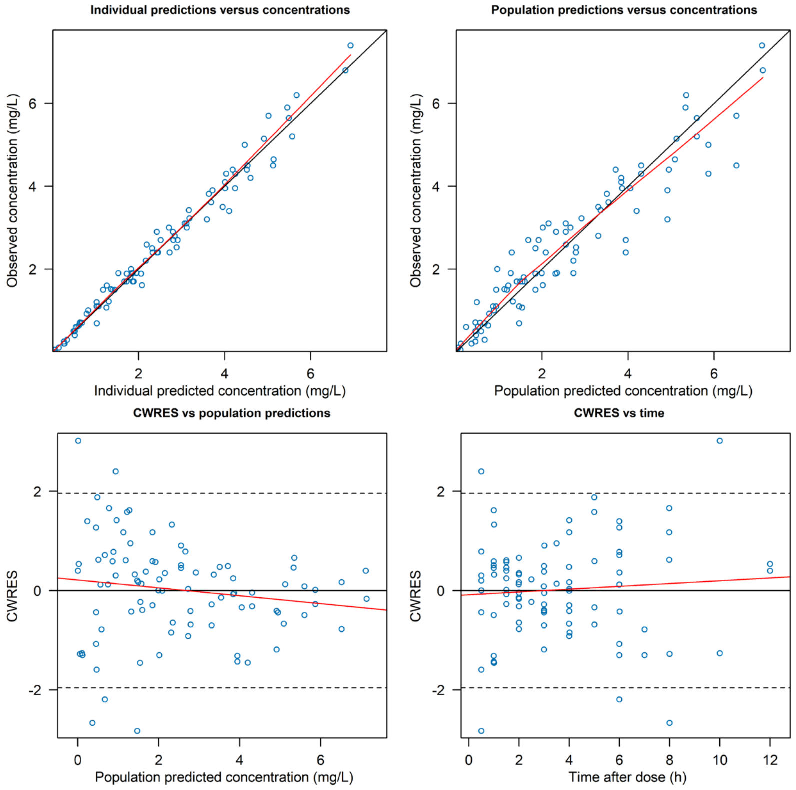

Figure S6. Goodness-of-fit plot for the final model following oral administration of tablet, crushed tablet and suspension to adults and children.

Open circles show individual data points. Solid red line is a trend line. CWRES, conditional weighted residuals.

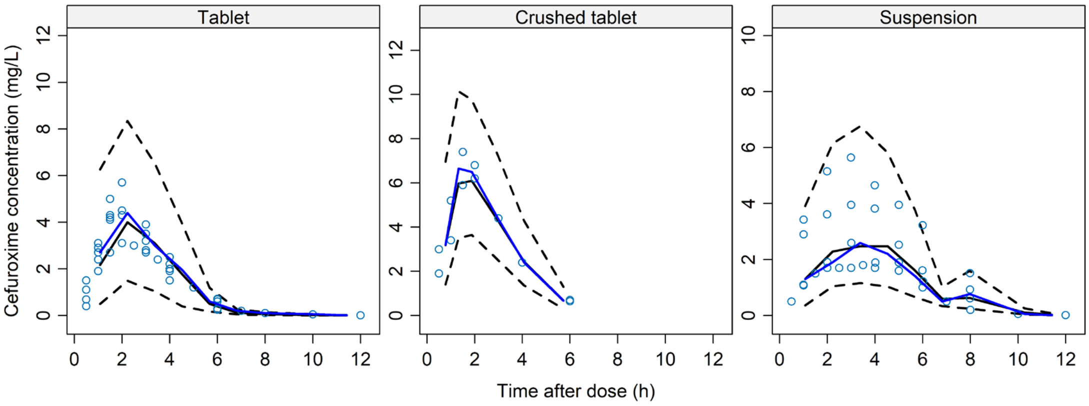

Figure S7. Visual predictive check for the final model following oral administration of tablet, crushed tablet and suspension to adults and children, stratified by formulation.

Blue circles and lines: observed concentrations and median; black solid and dotted lines: median and 90% prediction intervals.

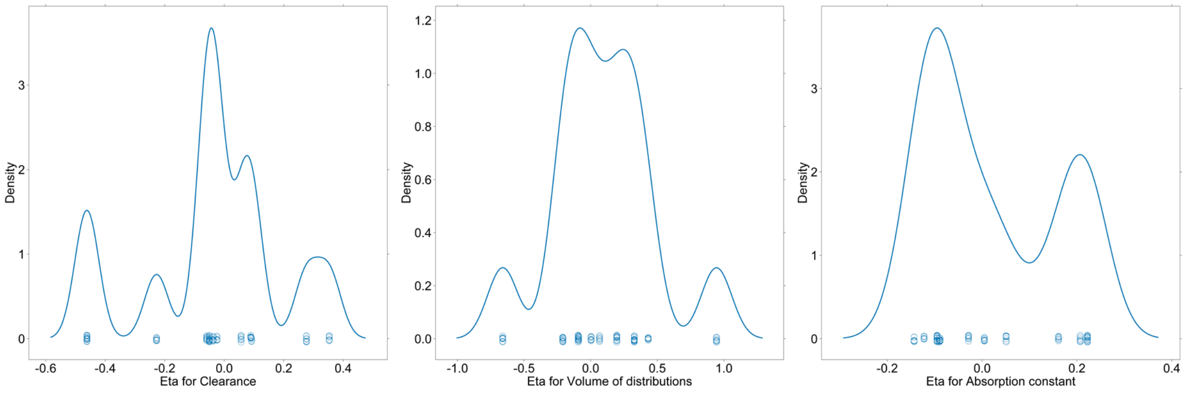

Figure S8. Density plots of eta distributions for CL, Vd and Ka from the final model following oral administration of tablet, crushed tablet and suspension to adults and children.

CL, clearance; Eta, NONMEM interindividual variability; Ka, absorption rate constant; Vd, volume of distribution.

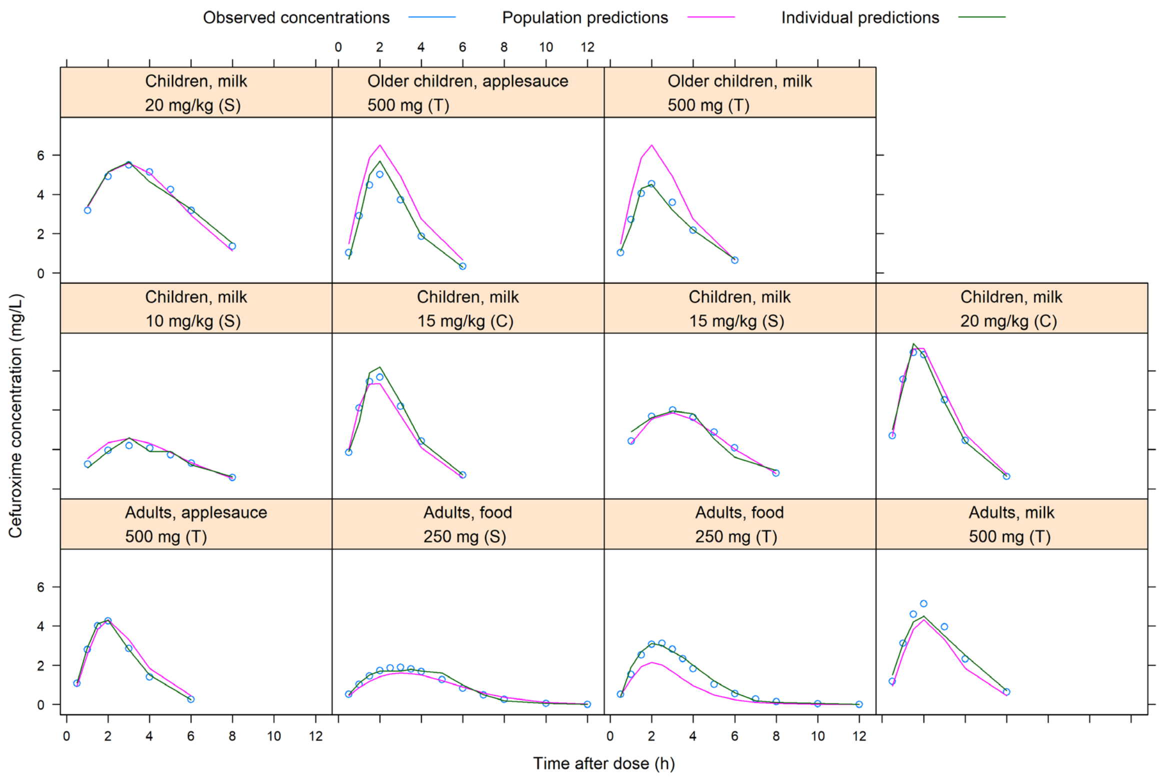

Figure S9. Observed (open circles, and predicted cefuroxime concentrations in plasma or serum stratified by study arm, and population (adult vs. children). Predicted cefuroxime concentrations simulated using the final model following oral administration of tablet, crushed tablet and suspension to adults and children.

T, tablet; C, crushed tablet; S, suspension.

Table S4. Reported and model-predicted mean estimates of $AUC_{0-\infty}$ and Cmax for cefuroxime along with the corresponding ratio between observed and predicted parameters.

Source	Cohort	Cmax (mg/L)			$AUC_{0-\infty}$ (mg×h/L)		
		Observed	Predicted	Ratio O/P	Observed	Predicted	Ratio O/P
Ginsburg, 1985 [1]	Adults 500 mg tablet (Milk)	4.5 (1.0)	4.3 (1.0)	1.05	16.9 (4.4)	13.6 (3.3)	1.24
	Adults 500 mg tablet (Apple sauce)	4.3 (1.3)	4.3 (1.1)	1.00	13.6 (3.7)	13.7 (3.6)	0.993
	Infants and children 15 mg/kg crushed tablet (Milk)	6.2 (2.1)	5.5 (1.4)	1.13	17.8 (4.9)	17.7 (4.5)	1.01
	Infants and children 20 mg/kg crushed tablet (Milk)	7.4 (1.7)	7.4 (1.8)	1.00	23.3 (4.1)	23.6 (5.8)	0.987
	Older children 500 mg tablet (Milk)	4.5 (2.8)	6.5 (1.7)	0.69	22.3 (7.9)	20.6 (5.4)	1.08
	Older children 500 mg tablet (Apple sauce)	5.7 (1.9)	6.5 (1.7)	0.88	16.5 (4.5)	20.5 (5.2)	0.805
Donn, 1994 [2]	Adults 250 mg tablet (Food)	3.83 (0.87)	2.2 (0.5)	1.74	12.69 (1.97)	7.3 (1.8)	1.74
	Adults 250 mg suspension (Food)	2.21 (0.35)	2.2 (0.5)	1.00	11.29 (1.85)	10.1 (2.5)	1.12
Powell, 1991 [3]	Infants and children 10 mg/kg suspension (Milk)	3.3 (0.8)	3.2 (0.8)	1.03	12.4 (2.5)	15.0 (3.7)	0.827
	Infants & Children 15 mg/kg suspension (Milk)	5.1 (1.4)	4.8 (1.2)	1.06	22.5 (9.3)	22.5 (5.5)	1.00
	Infants & Children 20 mg/kg suspension (Milk)	7.0 (2.0)	7.0 (1.8)	1.00	32.8 (10.2)	32.9 (8.5)	0.997

Point estimates are arithmetic means.

AUC, area under the concentration vs. time curve; Cmax, peak concentration; PK, pharmacokinetics; SD, standard deviation

3.3. IM/IV administration model

It was of interest to understand urinary tract exposure to cefuroxime in urinary tract infections. To characterise urinary cefuroxime exposure and enable the prediction of cefuroxime urine concentrations, a second model was built based on the cefuroxime concentrations in serum and cefuroxime amounts in urine from Foord et al. 1976 [4]. A diagram of the model structure is provided in **Fig. S10**. Serum concentrations after IM and IV administration were described using a dose compartment with first-order absorption for IM doses, a central compartment for serum, and two peripheral compartments. It is likely that these peripheral compartments could only be discerned in the IM/IV data, as the oral absorption phase often masks peripheral distribution from the concentration over time curve. By contrast, IV doses were delivered directly into the central compartment, with bioavailability fixed to 100%, whereas bioavailability of the IM doses was found to be comparable (i.e., 100%). The first-order elimination rate of cefuroxime from serum resulted in equivalent increases of amounts into the urinary tract compartment. A separate compartment was added to keep track of urine volume production (V5). Urine cefuroxime concentrations were predicted by dividing the urine cefuroxime amount by the urine volume produced over predefined time intervals. Interindividual variability was estimated to be small for clearance (CL) and central volume of distribution (V2), and was too small to be estimated for the absorption rate (Ka). However, this is a common finding following IV administration to healthy individuals; therefore these parameters were considered to adequately represent the overall interindividual variability in the patient population. All parameters were estimated with good precision (RSE<40%), as determined by the covariance step in NONMEM (**Table S5**).

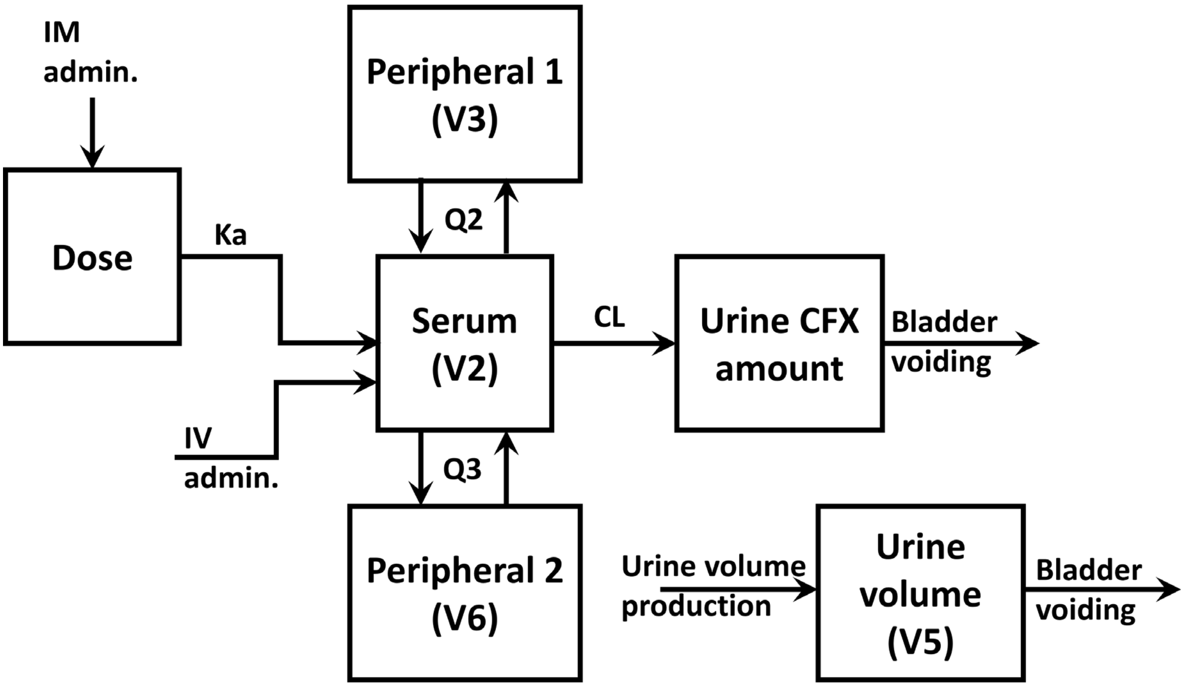

Figure S10. Diagram of the model structure describing the PK of cefuroxime after IM/IV administration of cefuroxime axetil.

CFX, cefuroxime; CL, clearance; IM, intramuscular; IV, intravenous.

The model described both serum and urine data well (**Fig. S11** and **Fig. S12**, respectively). Low variability in the data meant that interindividual variability was estimated to be minimal on disposition parameters, and this is reflected in the goodness-of-fit plots, as the population predictions show minor differences from the observed concentrations or amounts. Individual profiles show that the amount excreted in urine over time was well described by the model under the assumption that the bioavailable dose of cefuroxime did not end up anywhere else than in urine.

Table S5. Parameter estimates along with their precision (RSE%) and shrinkage for the IM/IV model.

Parameter	Value	RSE%	Shrinkage %
CL (L/h)	8.85	6	
Vd (L)	6.35	15	
Ka (h^{-1}) IM	1.71	9	
F1 IM	1.0	2	
Q2 (L/h)	17.2	12	
V3 (L)	5.28	10	
Q3 (L/h)	0.758	22	
V6 (L)	1.88	17	
Ω_{CL}	0.0114	35	0
Ω_{Vd}	0.0693	24	4
Σprop	0.0222	11	5

CL, clearance; F1, bioavailability; IM, intramuscular, IV, intravenous; Ka, absorption rate constant; RSE, relative standard error; Vd, volume of distribution; Ω_x, variance of parameter *x*; Σprop, proportional residual error.

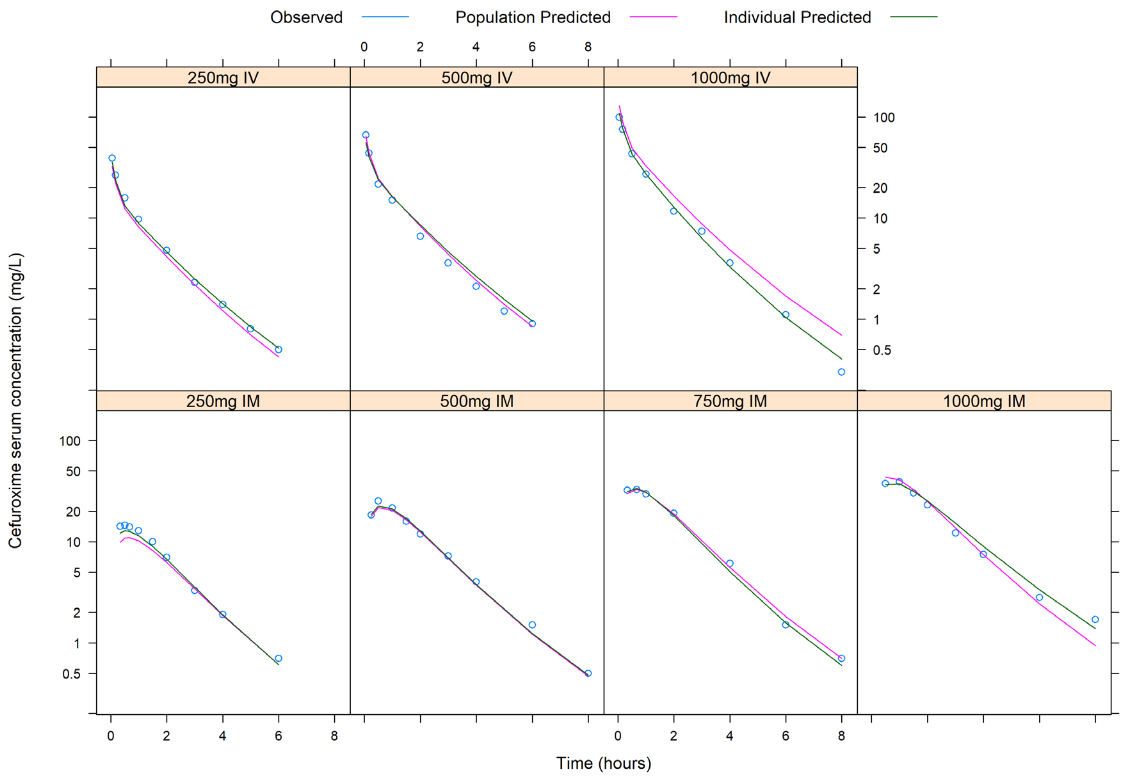

Figure S11. Observed and predicted cefuroxime concentrations in serum after IM or IV administration. Results are shown stratified by route of administration and dose level (treatment arm).

IM, intramuscular; IV, intravenous.

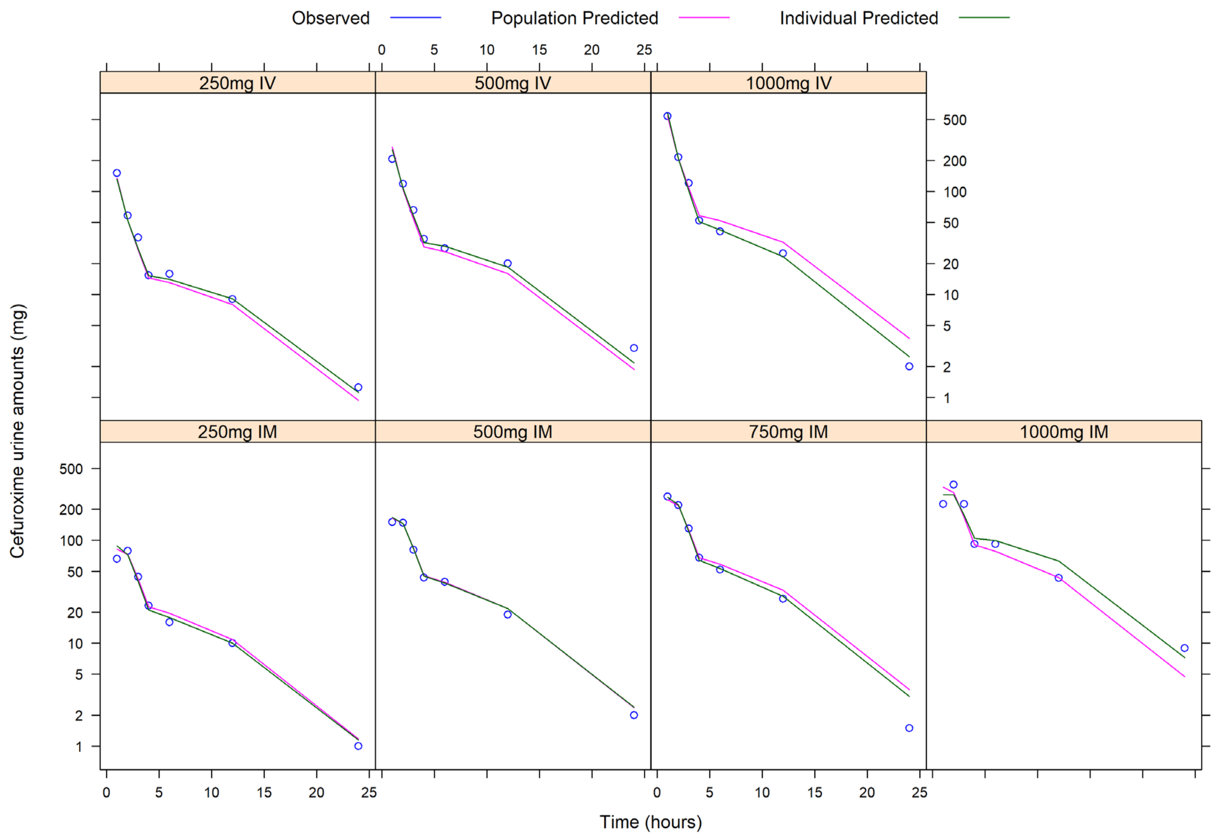

Figure S12. Observed and predicted cefuroxime amount in urine after IM or IV administration. Results are shown stratified by route of administration and dose level.

IM, intramuscular; IV, intravenous.

4. Prediction of cefuroxime concentrations

4.1. Scenario 1: Switching from suspension to tablet (patients weighing 20 – 40 kg)

Patients of weight 20–≤40 kg are more likely to use cefuroxime axetil as a suspension due to difficulties with swallowing tablets, but, due to some differences in bioavailability between suspension and tablet formulations, it is of interest to determine whether there is an impact of this difference on efficacy. Thus, the first simulation scenario was aimed at establishing the appropriateness of switching from suspension to tablet in patients weighing between 20–40 kg. The conversion from the different suspension doses (higher: 15 mg/kg twice daily [BID], lower: 10 mg/kg BID) to tablet doses is provided in **Table S6** given that only whole tablets can be given and tablets should not be crushed or split. Cefuroxime concentrations were simulated

for 1000 patients at each of the weights 20, 30 and 39 kg. Statistical summaries of AUC and Cmax, T>MIC and PTA are provided in **Table S8**, **S9,** and **S10,** respectively. Graphical summaries of the PTA vs. MICs are discussed in the main manuscript.

4.2. Scenario 2: Probability of target attainment for cefuroxime axetil doses in adults and children (systemic exposure)

In this scenario, concentration vs. time profiles of cefuroxime were simulated in patients across the following weight ranges: 4–6 kg, 6–12 kg, 12–20 kg, 20–40 kg, 40–60 kg, 60–80 kg, and 80–100 kg. A total of 10000 individuals were simulated, evenly and uniformly divided across the weight ranges, i.e. 1250 individuals per weight range category. Individual body weight for each patient was randomly assigned based on a uniform distribution within each group or category. The weight ranges of 4–6 kg, 6–12 kg and 12–20 kg were selected because these correspond to ranges relevant to suspension dosing guidance in the label, and 20–40 kg is the weight range where dosing guidance is provided separately. Finally, 40–60, 60–80 and 80–100 were selected as these follow the same 20 kg range as that of 20–40 kg, and correspond to the reasonable range of weights that the label provides guidance for. Weights >100 kg were not simulated as these are more likely to correspond to obese patients, for which no data was available during model building and thus it is uncertain if the model could accurately simulate the concentration vs time profiles in these patients. Likewise, simulations were not performed for pregnancy, or other special populations. Individual cefuroxime concentrations were simulated every 30 min over a 24-hour dosing interval for tablets of 250 and 500 mg BID (patients >40 kg), or suspension of 10 and 15 mg/kg BID (≤40 kg). Even though concentration vs time profiles were predicted after single doses, these results also reflect systemic and urine exposure after multiple doses. This is due to the relatively short elimination half-life of cefuroxime, which does not allow for accumulation following repeated dosing. Statistical summaries of AUC and Cmax, T>MIC and PTA are provided in **Tables S11**, **S12**, and **S13,** respectively. T>MIC and PTA were calculated based on the simulated concentrations, as discussed in the main manuscript. The simulated cefuroxime concentrations are shown in **Fig. S13 and S14,** for the lower and higher doses, respectively.

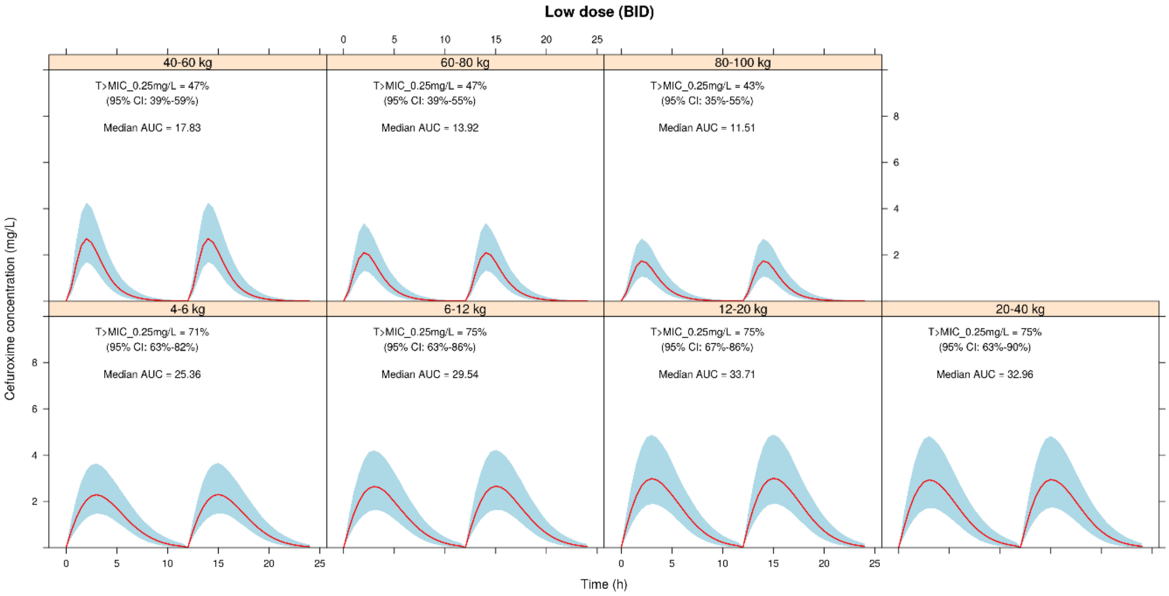

Figure S13. Predicted concentration vs time profile following cefuroxime doses of 10 mg/kg BID for ≤40kg and 250 mg BID for >40 kg, stratified by weight bands. Median AUC and time above MIC (T>MIC) along with the 95% confidence interval are summarised for MIC = 0.25 mg/mL. Curves depict median profiles and corresponding 95% prediction interval.

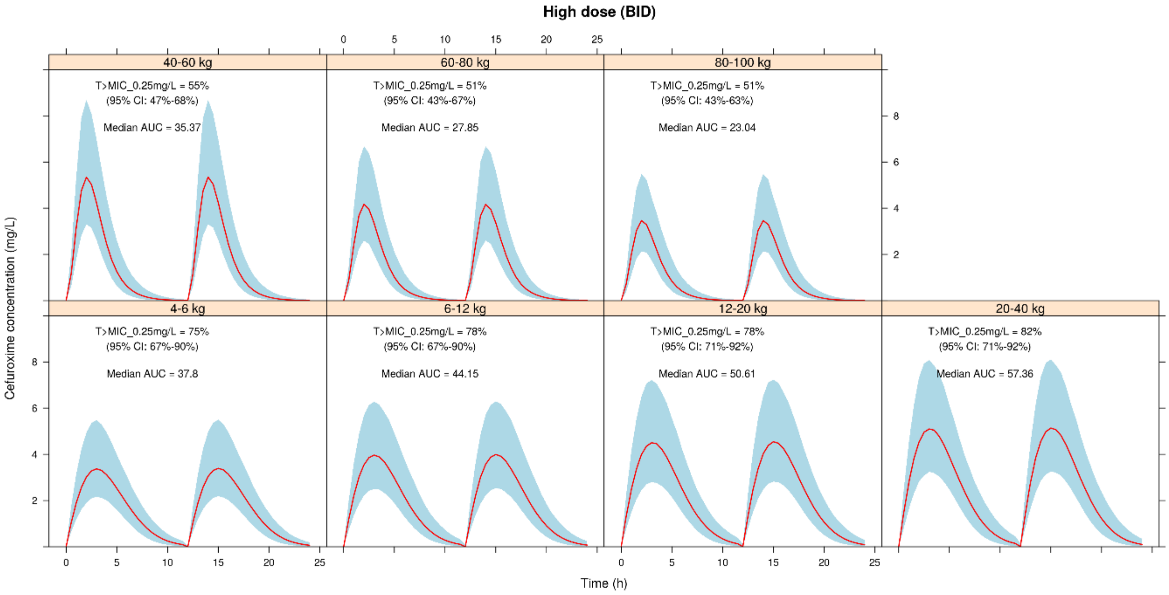

Figure S14. Predicted concentration vs time profile following cefuroxime doses of 15 mg/kg BID for ≤40kg and 500 mg BID for >40 kg, stratified by weight bands. Median AUC and time above MIC (T>MIC) along with the 95% confidence interval are summarised for MIC = 0.25 mg/mL. Curves depict median profiles and corresponding 95% prediction interval.

4.3. Scenario 3: Probability of target attainment for cefuroxime axetil doses in adults and children (urine exposure)

Two approaches were used to predict cefuroxime concentrations in urine over the dosing interval. First, simulations were performed based on the PK model describing urine excretion after IM/IV administration. Then, simulations were implemented using an extension of the PK model obtained after oral administration of the suspension and tablet dosage forms. This was deemed necessary as the model derived from IM/IV data included only adult subjects and extrapolation to the paediatric population was based on allometric scaling principles. In addition, the model derived from oral data after administration of suspension and tablets provided details on the rate and nonlinearity in the absorption of cefuroxime suspension, which could not be inferred from IM data. Together the two models provided a means to assess the accuracy of extrapolated results and overall model performance in the virtual population cohort.

Cefuroxime concentrations in urine were predicted for patients of 10, 20, 40 and 70 kg. In this setting, patients of weight 10, 20 and 40 kg were simulated to receive suspension (10 or 15 mg/kg capped to 250 mg or 500 mg, respectively), and those of weight 70 kg received tablet doses (250 or 500 mg). The predicted cefuroxime concentrations in urine by each model were compared graphically, and T>MIC and probability of target attainment were determined against the most prevalent MIC for *E. coli* (European Committee on Antimicrobial Susceptibility Testing MIC required to inhibit the growth of 90% of organisms (8 mg/L) [13]. Statistical summaries of AUC and Cmax, T>MIC and PTA are provided in **Tables S14**, **S15**, and **S16,** respectively. T>MIC and PTA were calculated based on the simulated concentrations, as discussed in the main manuscript.

4.4. Predicting urine cefuroxime concentrations using the IM/IV model, adjusted for bioavailability of suspension and tablet

Urinary cefuroxime concentrations following administration of suspension or tablet doses were simulated using the IM/IV PK model after adjustment for the differences in bioavailability. For tablets, bioavailability has been previously established to be approximately 50% [14], whereas the bioavailability of the suspension has been shown to be roughly 80% of that of tablets, or 40% in absolute terms [2]. Thus, F1 was set to reflect the oral dosage forms, with values for tablets set to 50% and suspension to 40%. Given the focus of this simulation scenario on urinary excretion, no further adaptations were made to the

model. It was assumed that differences in the absorption rate and overall absorption profile between IM and oral administration would have minor impact on the rate of elimination (i.e. first-order process, no flip-flop kinetics) and consequently on the total amount excreted over the dosing interval. In addition, urine volume production was set to those previously described in **Section 2 – Model building**. Urine concentrations were calculated by dividing the urine cefuroxime amount by the predicted urine volume produced over the same interval [13].

4.5. Predicting urine cefuroxime concentrations using an adapted model for tablet and suspension

To assess the potential effect of slower absorption on the overall time course of cefuroxime concentrations in urine and explore consistency in the simulated data, urine compartments were added to the model previously developed to describe the time course of cefuroxime concentrations in serum after oral administration of suspension and tablets. Based on the learnings from the IM/IV model, that all systemic cefuroxime seems to be eliminated directly into urine, simulations were then performed assuming direct elimination of cefuroxime into a urine amount compartment. Urine volume production was set to those previously described in **Section 2 – Model building**. Urine concentrations were calculated by dividing the urine cefuroxime amount by the predicted urine volume produced over the same interval.

In addition to the inclusion of relevant urine compartments, model estimates describing the (apparent) clearance were further adjusted to account for oral bioavailability. Thus, a bioavailability term (F_{urine}) was used to ensure accurate estimation of the actual fraction of cefuroxime excreted into urine following administration of tablets and suspension. As such, F_{urine} was set to 50% for tablets and 40% for suspension (see Section 4.4 above) [2, 14]. This allowed simulations of both serum/plasma and urine concentrations taking into account the mass balance between compartments. Consequently, this yielded a maximum of 50% and 40% of the oral dose being recovered in urine after administration of tablet and suspension, respectively. A comparison of the typical cefuroxime urine concentration vs, time profile from the adapted IM/IV model and adapted oral administration model is provided in **Fig. 4** in the main manuscript.

Table S6. Dose conversion from suspension to tablets in patients with body weight 20–40 kg, who are able to swallow tablets of 250 mg.

WT	Daily 'higher' dose for suspension	Converted tablet 'higher' daily dose	Daily 'lower' dose for suspension	Lower maximum daily dose
20 kg	20 kg x 15 mg/kg BID = 600 mg	2 tablets of 250 mg (1x AM, 1x PM) = 500 mg	20 kg x 10 mg/kg BID = 400 mg	2 tablets of 250 mg (1x AM, 1x PM) = 500 mg
30 kg	30 kg x 15 mg/kg BID = 900 mg	3 tablets of 250 mg (2x AM, 1x PM) = 750 mg	30 kg x 10 mg/kg BID = 600 mg (capped to 500 mg)	2 tablets of 250 mg (1x AM, 1x PM) = 500 mg
39 kg	39 kg x 15 mg/kg BID = 1170 mg (capped to 1000 mg)	4 tablets of 250 mg (2x AM, 2x PM) =1000 mg	39 kg x 10 mg/kg BID = 780 mg (capped to 500 mg)	2 tablets of 250 mg (1x AM, 1x PM) = 500 mg

BID, twice daily; WT, weight.

Table S7. Cefuroxime axetil indications, common causative pathogens, CLSI and EUCAST MIC susceptibility breakpoints.

Indication	Most common causative pathogen	CLSI MIC susceptibility breakpoints (mg/L)	EUCAST MIC susceptibility breakpoints (mg/L)
Acute tonsillitis and pharyngitis	*S. pyogenes*	≤0.12[a]	≤0.25[b]
Acute otitis media	*S. pneumoniae*	≤1	≤0.25
Acute bacterial sinusitis	*S. pneumoniae*	≤1	≤0.25
	H. influenzae	≤4	≤0.001
Community-acquired pneumonia	*S. pneumoniae*	≤1	≤0.25
Acute exacerbation of chronic obstructive pulmonary disease	*S. pneumoniae*	≤1	≤0.25
Cystitis	*Enterobacterales (E. coli)*	≤4[c]	≤8[d,e]
Pyelonephritis	*Enterobacterales (E. coli)*	≤4[c]	≤8[d,e]
Skin and soft tissue infections	*S. aureus*	Note[f]	Note[g]

[a]Penicillin MIC susceptibility breakpoint captured in the table. An organism that is susceptible to penicillin can be considered susceptible to cefuroxime when used for approved indications and does not need to be tested against those agents. For group A β-hemolytic streptococci, penicillin is also a surrogate for cefuroxime.
[b]Benzylpenicillin MIC susceptibility breakpoint captured in the table. The susceptibility of *Streptococcus* groups A, B, C and G to cephalosporins is inferred from the benzylpenicillin susceptibility.
[c]Breakpoints are for cefazolin when used as a surrogate test to predict results for oral cefuroxime when used for therapy of uncomplicated UTIs due to *E. coli*, *K. pneumoniae*, and *P. mirabilis*. Cefazolin tested as a surrogate may overcall resistance to cefuroxime. If cefazolin tests resistant, test cefuroxime individually if needed for therapy.
[d]The cephalosporin breakpoints for *Enterobacterales* will detect all clinically important resistance mechanisms (including ESBL and plasmid mediated AmpC).
[e]Uncomplicated UTI only, *E. coli*, *Klebsiella* spp. (except *K. aerogenes*), *Raoultella* spp. and *P. mirabilis*.
[f]Oxacillin (or cefoxitin) results can be applied to the other penicillinase-stable penicillins. For agents with established clinical efficacy and considering site of infection and appropriate dosing, methicillin (oxacillin)-susceptible staphylococci can be considered susceptible to cefuroxime. Thus, the susceptibility breakpoint for oxacillin against methicillin susceptible *S. aureus* (MIC≤2 mg/L) would apply for cefuroxime axetil.
[g]Susceptibility of staphylococci to cephalosporins is inferred from the cefoxitin susceptibility except for cefixime, ceftazidime, ceftazidime-avibactam, ceftibuten, and ceftolozane-tazobactam, which do not have breakpoints and should not be used for staphylococcal infections. Isolates that test susceptible to benzylpenicillin and cefoxitin can be reported susceptible to all penicillins. Susceptibility breakpoint for benzylpenicillin against *S. aureus i*s MIC≤0.125 mg/L although specific breakpoint for cefuroxime axetil is not provided.

CLSI, Clinical and Laboratory Standards Institute; EUCAST, European Committee on Antimicrobial Susceptibility Testing; MIC, minimum inhibitory concentration; UTI, urinary tract infection.

Table S8. AUC and Cmax in plasma following administration of lower (10 mg/kg) and higher (15 mg/kg) doses of cefuroxime, as implemented in simulation scenario 1. Values are mean (SD).

Body weight (kg)		Suspension		Tablet*	
		AUC (mg×h/L)	Cmax (mg/L)	AUC (mg×h/L)	Cmax (mg/L)
Lower dose	20	46.2 (10.4)	4.1 (1.8)	36.1 (8.3)	5.5 (1.2)
	30	34.7 (8.0)	3.1 (0.7)	26.9 (6.1)	4.1 (0.9)
	39	28.5 (6.1)	2.5 (0.6)	22.3 (4.7)	3.4 (0.7)
Higher dose	20	55.9 (12.4)	5.0 (1.1)	36.2 (8.4)	5.5 (1.3)
	30	61.9 (13.3)	5.5 (1.2)	40.7 (9.2)	8.2 (1.9)
	39	56.6 (13.0)	5.0 (1.2)	43.8 (9.8)	6.6 (1.5)

*Tablet doses rounded to the nearest whole tablet

Table S9. T>MIC based on plasma cefuroxime concentrations, as implemented in simulation scenario 1. Values are mean (SD).

Body weight (kg)		Suspension		Tablet*	
		MIC = 0.25	MIC = 0.5	MIC = 0.25	MIC = 0.5
Lower dose	20	82.2 (6.6)	73.0 (6.3)	57.2 (5.9)	49.2 (5.1)
	30	78.9 (6.5)	69.2 (6.1)	54.0 (5.8)	45.9 (4.9)
	39	76.4 (6.4)	65.9 (5.8)	52.0 (5.4)	43.6 (4.6)
Higher dose	20	84.4 (6.9)	75.7 (6.8)	57.0 (6.0)	49.2 (5.2)
	30	85.8 (6.3)	77.3 (6.3)	57.8 (6.0)	49.8 (5.2)
	39	84.9 (6.5)	76.3 (6.5)	59.7 (6.3)	51.7 (5.4)

*Tablet doses rounded to the nearest whole tablet. MIC, minimum inhibitory concentration (mg/L)

Table S10. Probability of target attainment based on plasma cefuroxime concentrations, as implemented for simulation scenario 1.

Body weight (kg)		Suspension		Tablet*	
		MIC = 0.25	MIC = 0.5	MIC = 0.25	MIC = 0.5
Lower dose	20	100%	100%	100%	99.3%
	30	100%	100%	100%	95.5%
	39	100%	100%	99.7%	87.0%
Higher dose	20	100%	100%	100%	99.0%
	30	100%	100%	100%	98.8%
	39	100%	100%	100%	99.8%

*Tablet doses rounded to the nearest whole tablet. MIC, minimum inhibitory concentration (mg/L)

Table S11. AUC and Cmax in plasma, as implemented in simulation scenario 2. Values are mean (SD).

Weight bands	Lower dose		Higher dose	
	AUC (mg×h/L)	Cmax (mg/L)	AUC (mg×h/L)	Cmax (mg/L)
4 – 6 kg	26.3 (6.1)	2.4 (0.6)	39.1 (8.9)	3.5 (0.8)
6 – 12 kg	30.6 (7.3)	2.7 (0.7)	45.8 (11.0)	4.1 (1.0)
12 – 20 kg	34.9 (8.1)	3.1 (0.8)	52.5 (12.3)	4.7 (1.1)
20 – 40 kg	34.2 (8.5)	3.1 (0.8)	59.1 (13.8)	5.3 (1.3)
40 – 60 kg	18.5 (4.5)	2.8 (0.7)	37.0 (9.0)	5.6 (1.4)
60 – 80 kg	14.5 (3.4)	2.2 (0.5)	29.0 (7.0)	4.3 (1.0)
80 – 100 kg	11.9 (2.8)	1.8 (0.4)	24.0 (5.8)	3.6 (0.8)

Table S12. T>MIC based on plasma cefuroxime concentrations, as implemented in simulation scenario 2. Values are mean (SD).

Weight bands	Lower dose		Higher dose	
	MIC = 0.25	MIC = 0.5	MIC = 0.25	MIC = 0.5
4 – 6 kg	74.3 (6.0)	63.7 (5.7)	79.7 (6.3)	70.3 (5.9)
6 – 12 kg	76.8 (6.3)	66.7 (5.9)	81.5 (6.6)	72.5 (6.3)
12 – 20 kg	78.8 (6.4)	69.0 (6.0)	83.7 (6.4)	74.9 (6.1)
20 – 40 kg	78.2 (6.4)	68.3 (6.1)	85.0 (6.6)	76.4 (6.6)
40 – 60 kg	50.0 (5.4)	40.8 (5.0)	57.9 (6.3)	50.0 (5.5)
60 – 80 kg	47.3 (5.0)	36.5 (4.9)	55.4 (5.8)	47.2 (5.0)
80 – 100 kg	44.7 (5.0)	33.4 (4.8)	53.3 (6.0)	44.8 (5.1)

MIC, minimum inhibitory concentration (mg/L)

Table S13. Probability of target attainment based on plasma cefuroxime concentrations, as implemented for simulation scenario 2.

Weight bands	Lower dose		Higher dose	
	MIC = 0.25	MIC = 0.5	MIC = 0.25	MIC = 0.5
4 – 6 kg	100%	100%	100%	100%
6 – 12 kg	100%	100%	100%	100%
12 – 20 kg	100%	100%	100%	100%
20 – 40 kg	100%	100%	100%	100%
40 – 60 kg	99.3%	66.0%	100%	99.6%
60 – 80 kg	97.7%	31.3%	100%	97.4%
80 – 100 kg	89.8%	11.7%	99.9%	90.1%

MIC, minimum inhibitory concentration (mg/L)

Table S14. AUC and Cmax in urine, as implemented in simulation scenario 3. Values are mean (SD)

Weight band	Lower dose		Higher dose	
	AUC (mg×h/L)	Cmax (mg/L)	AUC (mg×h/L)	Cmax (mg/L)
4 – 6 kg	2192.8 (22.4)	346.8 (26.7)	3287.3 (33.0)	521.3 (40.4)
6 – 12 kg	2194.3 (22.2)	347.6 (26.0)	3290.3 (34.0)	519.9 (39.7)
12 – 20 kg	2831.3 (505.8)	417.0 (64.3)	4264.9 (754.5)	629.9 (97.0)
20 – 40 kg	2972.1 (330.7)	424.9 (57.2)	5200.1 (700.1)	742.0 (112.9)
40 – 60 kg	2947.9 (354.0)	703.7 (99.0)	5903.1 (726.8)	1410.2 (198.1)
60 – 80 kg	2437.2 (88.2)	576.9 (46.5)	4864.5 (174.4)	1156.4 (97.6)
80 – 100 kg	2445.9 (88.7)	573.7 (44.5)	4882.9 (174.5)	1150.5 (94.3)

Table S15. T>MIC based on urine cefuroxime concentrations, as implemented in simulation scenario 3. Values are mean (SD).

Weight band	Lower dose		Higher dose	
	MIC = 8	MIC = 16	MIC = 8	MIC = 16
4 – 6 kg	99.6 (2.4)	96.0 (6.4)	99.9 (1.2)	98.9 (3.9)
6 – 12 kg	99.6 (2.3)	96.1 (6.4)	99.9 (1.0)	98.9 (3.8)
12 – 20 kg	99.9 (1.3)	98.6 (4.2)	100 (0.4)	99.6 (2.3)
20 – 40 kg	100 (0)	100 (0)	100 (0.0)	100 (0.0)
40 – 60 kg	99.4 (3.2)	94.2 (7.9)	100 (0.7)	99.3 (3.4)
60 – 80 kg	98.8 (4.4)	91.9 (8.3)	99.9 (1.2)	98.6 (4.6)
80 – 100 kg	98.9 (4.1)	92.4 (8.3)	99.9 (1.2)	98.9 (4.1)

MIC, minimum inhibitory concentration (mg/L)

Table S16. Probability of target attainment based on urine cefuroxime concentrations, as implemented for simulation scenario 3.

Weight band	Lower dose		Higher dose	
	MIC = 0.25	MIC = 0.5	MIC = 0.25	MIC = 0.5
4 – 6 kg	100%	100%	100%	100%
6 – 12 kg	100%	100%	100%	100%
12 – 20 kg	100%	100%	100%	100%
20 – 40 kg	100%	100%	100%	100%
40 – 60 kg	100%	100%	100%	100%
60 – 80 kg	100%	100%	100%	100%
80 – 100 kg	100%	100%	100%	100%

MIC, minimum inhibitory concentration (mg/L)

References

1. Ginsburg CM, McCracken GH, Petruska M, Olson K. Pharmacokinetics and bactericidal activity of cefuroxime axetil. Antimicrob Agents Chemother 1985; 28(4):504–7.
2. Donn KH, James NC, Powell JR. Bioavailability of cefuroxime axetil formulations. J Pharm Sci 1994; 83(6):842–4.
3. Powell DA, James NC, Ossi MJ, Nahata MC, Donn KH. Pharmacokinetics of cefuroxime axetil suspension in infants and children. Antimicrob Agents Chemother 1991; 35(10):2042–5.
4. Foord RD. Cefuroxime: human pharmacokinetics. Antimicrob Agents Chemother 1976; 9(5):741–7.
5. Luscombe M, Owens B. Weight estimation in resuscitation: is the current formula still valid? Arch Dis Child 2007; 92:412–5.
6. Bullita JB, Landersdorfer CB, Kinzig M, Holzgrabe U, Sorgel F. New semiphysiological absorption model to assess the pharmacodynamic profile of cefuroxime axetil using nonparametric and parametric population pharmacokinetics. Antimicrob Agents Chemother 2009; 53(8):3462–71.
7. Israr F, Mahmood ZA, Hassan F, Hasan SMF. Pharmaceutical evaluation of cefuroxime axetil tablets available in drug market of Pakistan. Indian J Pharm Sci 2016; 78(1):17–26.
8. D'Agate S, Musuamba FT, Della Pasqua O. Dose rationale for amoxicillin in neonatal sepsis when referral is not Possible. Front Pharmacol. 2020; 11:521933.
9. D'Agate S, Musuamba FT, Jacqz-Aigrain E, Della Pasqua O. Simplified Dosing Regimens for Gentamicin in Neonatal Sepsis. Front Pharmacol. 2021; 12:624662.
10. Mahmood I. Dosing in children: a critical review of the pharmacokinetic allometric scaling and modelling approaches in paediatric drug development and clinical settings. Clin Pharmacokinet 2014; 53(4):327–46.
11. De Cock PAJG, van Dijkman SC, de Jaeger A, Willems J, Carlier M, Verstraete AG, et al. Dose optimization of piperacillin/tazobactam in critically ill children. J Antimicrob Chemother 2017; 72(7):2002–11.
12. Weingartner L, Sitka U, Patsch R, Richter I. Experience with amoxycillin in neonates and premature babies. Int J Clin Pharmacol Biopharm 1977; 15 (4):184–88.
13. Hazinski MF. Nursing Care of the Critically Ill Child, St. Louis: Mosby; 1992.

14. Finn A, Straughn A, Meyer M, Chubb J. Effect of dose and food on the bioavailability of cefuroxime axetil. Biopharm Drug Dispos. 1987; 8(6):519-26.
